# Interferon-mediated NK cell activation increases cytolytic activity against T follicular helper cells and limits antibody response to SARS-CoV-2

**DOI:** 10.1038/s41590-025-02341-1

**Published:** 2025-11-21

**Authors:** Izumi de los Rios Kobara, Radeesha Jayewickreme, Madeline J. Lee, Aaron J. Wilk, Stanford COVID-19 Biobank, Madeline J. Lee, Aaron J. Wilk, Andra L. Blomkalns, Kari C. Nadeau, Samuel Yang, Angela J. Rogers, Catherine A. Blish, Andra L. Blomkalns, Kari C. Nadeau, Samuel Yang, Angela J. Rogers, Catherine A. Blish

**Affiliations:** 1 Department of Medicine, Stanford University School of Medicine, Stanford, CA USA; 2 Stanford Immunology Program, Stanford University School of Medicine, Stanford, CA USA; 3 Stanford Medical Scientist Training Program, Stanford University School of Medicine, Stanford, CA USA; 4 Department of Emergency Medicine, Stanford University School of Medicine, Stanford, CA USA; 5 Department of Environmental Health, Harvard School of Public Health, Boston, MA USA; 6 Department of Medicine and Division of Allergy and Inflammation, Beth Israel Deaconess Hospital, Boston, MA USA; 7 Division of Pulmonary, Allergy and Critical Care Medicine, Stanford University, Stanford, CA USA; 8 Chan Zuckerberg Biohub, San Francisco, CA USA

**Keywords:** Infectious diseases, Innate lymphoid cells, Antibodies

## Abstract

Natural killer (NK) cells are innate lymphocytes known for their ability to kill infected or malignant cells, but they have an overlooked role in regulating antibody responses. In mice, NK cells can kill T follicular helper (T_FH_) cells, decreasing somatic hypermutation and antibody titers. Although human NK cell activation correlates with poor vaccine response, the mechanisms of NK cell regulation of adaptive immunity in humans are poorly understood. Here we found that, in ancestral severe acute respiratory syndrome coronavirus 2 infection, individuals with the broadest neutralization profile had fewer NK cells that expressed inhibitory and immaturity markers, whereas NK cells from narrow neutralizers were highly activated and expressed interferon-stimulated genes. ISG-mediated activation in NK cells from healthy donors increased cytotoxicity toward induced T_FH_-like cells via NKG2D and NKp30. This work reveals that NK cell activation and dysregulated inflammation play a role in poor antibody response to severe acute respiratory syndrome coronavirus 2 and opens exciting avenues for designing improved vaccines and adjuvants to target emerging pathogens.

## Abstract (web summary)

In ancestral SARS-CoV-2 infection, NK cell interferon-stimulated gene (ISG)-driven activation correlated with poor antibody breadth. In vitro, NK cell ISG expression boosts cytotoxicity toward T_FH_-like cells, suggesting a mechanism for impaired antibody responses.

## Main

Severe acute respiratory syndrome coronavirus 2 (SARS-CoV-2) infection results in mild-to-fatal respiratory illness and has caused over 7 million deaths since the coronavirus disease 2019 (COVID-19) pandemic began in 2020 (refs. ^1,2^). Many individuals infected with SARS-CoV-2 develop antibodies that neutralize multiple variants; however, the breadth and potency of antibody repertoires vary across human individuals^3,4^. We define breadth as the ability of an individual’s antibody repertoire to neutralize multiple viral variants to which the individual has not been exposed. In SARS-CoV-2 infection, the antibody repertoire that results from infection or vaccination with the ancestral strain (Wuhan -1 (Wu-1)) has variable ability to neutralize variants of concern that evolved later in the pandemic such as alpha, beta, delta and omicron^5,6^. Factors that influence antibody breadth, particularly against variants to which the individual has not been exposed, are poorly understood. Here, we sought to identify features of the host peripheral immune response to ancestral SARS-CoV-2 infection that correlate with antibody breadth against future variants to inform our understanding of how to develop improved vaccines against coronaviruses and other emerging pathogens.

Natural killer (NK) cells are understudied regulators of antibody responses^7–18^. Classically known as innate lymphocytes that kill infected or malignant cells, NK cells also play immunoregulatory roles during viral infection owing to their interactions with other immune cells^14^. In mice, NK cells can kill T follicular helper (T_FH_) cells in the germinal center, which limits somatic hypermutation and decreases antibody titer in the setting of vaccination^12^. In vitro, NK cells can kill activated CD8^+^ T cells^19–21^, CD4^+^ T cells^15,22,23^ and dendritic cells^24,25^ and directly interact with B cells^26,27^. Human clinical trials for yellow fever, malaria and hepatitis B vaccines have identified NK cells as correlates of nonresponse. In each trial, higher antibody titers or protection from live pathogen challenge was associated with lower NK cell activation or reduced expression of NK cell functional gene modules^10,11,18^. Conversely, in the context of infection, dysfunctional NK cells are associated with broadly neutralizing antibodies in individuals with untreated, chronic human immunodeficiency virus infection^28^. NK cells can also play a positive role in the antibody response; NK cell secretion of interferon γ (IFNγ) is critical to the efficacy of the AS01 adjuvant in mice^9^. The complex roles of NK cells in immunity during vaccination and infection have yet to be fully elucidated and remain key areas for future research to unlock favorable human antibody responses.

To determine if NK cell phenotype correlates with antibody breadth in SARS-CoV-2 infection, we used publicly available data collected by our laboratory in March to June 2020. This dataset contains peripheral blood profiling of SARS-CoV-2-positive participants across the disease severity spectrum by single-cell RNA sequencing (scRNA-seq) and mass cytometry by time of flight (CyTOF)^29,30^. Matched serum was used to evaluate the antibody repertoire, enabling us to capture immune correlates of breadth in individuals from across the severity spectrum. We examined systemic immune differences between broad and narrow neutralizers and found major differences in the NK cell compartment. NK cells from narrow neutralizers were highly activated and expressed markers of cytotoxicity, including many IFN-stimulated genes (ISGs). Finally, we validated in vitro that ISG-driven activation in NK cells resulted in greater inflammatory responses, specific killing of induced T_FH_-like (iT_FH_-like) cells and suppression of B cell responses. Overall, this study demonstrates that NK cell phenotype is strongly correlated with neutralization breadth in COVID-19 and suggests that ISG-driven inflammation may contribute to poor antibody breadth.

## Results

### Cohort description

**Fig. 1:**
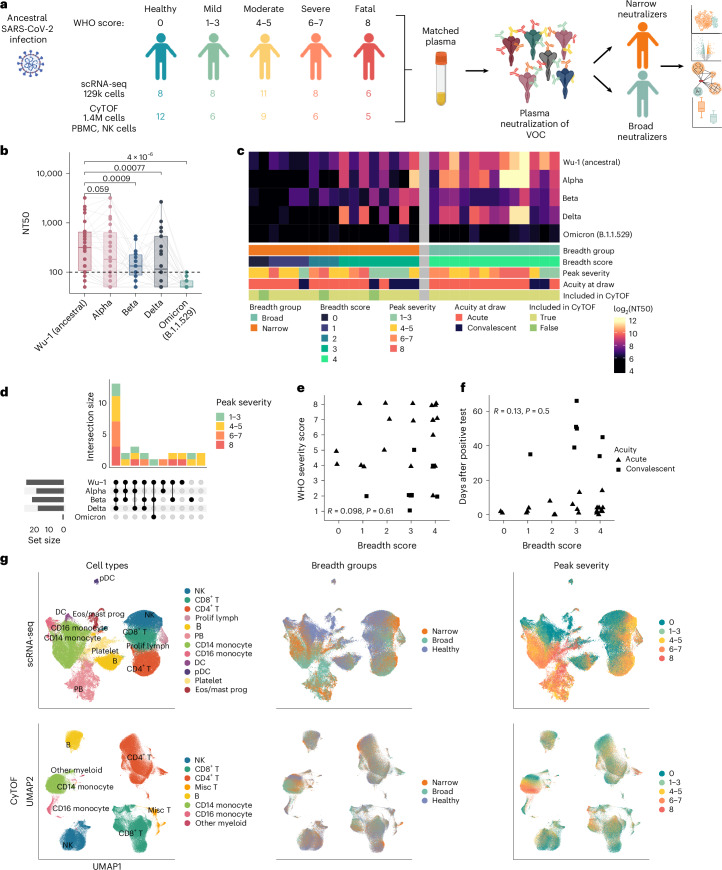
Heterogeneity in neutralization breadth against variants of concern after ancestral SARS-CoV-2 infection. **a**, Study outline summarizing number of samples profiled and peak WHO severity score for each modality. VOC, variant of concern **b**, Boxplot of NT50 against five SARS-CoV-2 variant pseudoviruses. Each point represents the average of three technical replicates. Boxplots are drawn as median (center line), interquartile range (IQR; box) and 1.5× IQR (whiskers). *P* values calculated using two-sided, paired Wilcoxon signed-rank test with Bonferroni’s correction for multiple hypothesis testing. Lines connect unique donors. **c**, Heatmap of NT50 against five variants for each participant (column). Each cell represents the average of three technical replicates. **d**, UpSet plot of all unique combinations of SARS-CoV-2 variants neutralized, colored by peak WHO severity score. **e**, Scatter plot of breadth score and WHO severity score. **f**, Scatter plot breadth score and days after positive test. *P* value for **e** and **f** calculated using two-sided Spearman rank correlation. Each point represents one donor and is shaped by sample acuity. **g**, UMAPs of complete scRNA-seq (top) and CyTOF (bottom) datasets colored by cell types, breadth groups and peak WHO severity score. PB, plasmablast; Eos, eosinophil; Prog, progenitor; Prolif lymph, proliferating lymphocyte. Panel **a** created with BioRender.com. Source data

**Extended Data Fig. 1:**
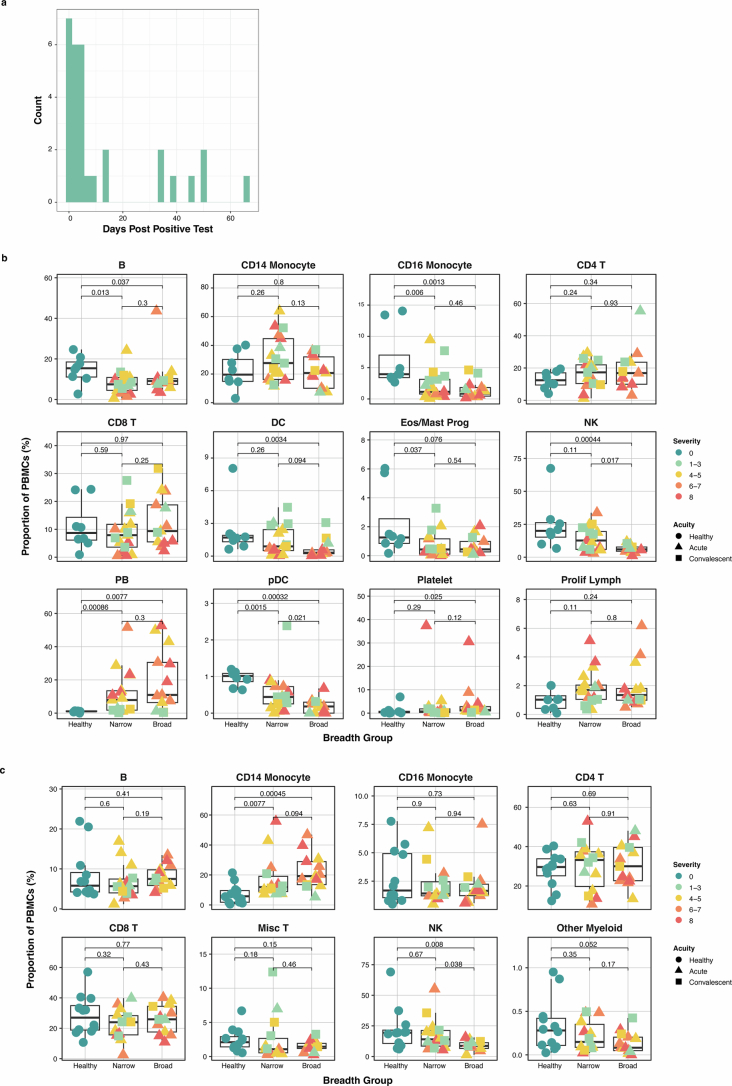
Cell Type Frequencies Across Breadth Groups in scRNA-seq and CyTOF. **a**. Histogram of days post positive test for each sample. **b**. Boxplots of cell type proportions from scRNA-seq data set in each breadth group. Platelets are excluded from the proportion calculations because their presence is related to sample processing.PB = plasmablast, Eos = eosinophil; Prog = progenitor; Prolif Lymph = proliferating lymphocyte. **c**. Boxplots of cell type proportions from CyTOF data colored by peak WHO severity score and shaped by acuity. All boxplots are drawn as median (center line), IQR (box), and 1.5x IQR (whiskers), colored by peak WHO severity score, and shaped by acuity. P-values by two-sided Wilcoxon rank sum test with Bonferroni’s correction for multiple hypothesis testing. Each point represents one donor.

Our laboratory previously profiled 30 participants with COVID-19 from across the severity spectrum and 7 healthy controls by scRNA-seq as well as 26 participants (a subset of the 30 in scRNA-seq) and 12 healthy controls by CyTOF^29,30^ (Fig. 1a). This cohort was well controlled for both viral variant and prior exposure to SARS-CoV-2, as these samples were collected during the first 4 months of the COVID-19 pandemic. Thus, all individuals experienced primary infection with ancestral SARS-CoV-2 because variants of concern had not yet evolved^4^. Peripheral blood mononuclear cells (PBMCs) and red blood cell-lysed whole blood were profiled using scRNA-seq, and PBMCs and NK cells were profiled using CyTOF (Supplementary Table 3). This cohort contained participants with COVID-19 from across the full range of the World Health Organization (WHO) disease severity spectrum, including participants with mild disease who remained unhospitalized (WHO 1–3), hospitalized participants with moderate disease (WHO 4–5), hospitalized participants with severe disease who required intubation (WHO 6–7) and participants who were later deceased (WHO 8). Samples were collected 0–66 days after a positive COVID-19 polymerase chain reaction (PCR) test (median 4 days), with the majority of samples drawn during the acute phase of disease (Extended Data Fig. 1a).

### Variability in antibody breadth during ancestral SARS-CoV-2 infection is independent of severity

To determine immunological correlates of antibody breadth in SARS-CoV-2 infection, we evaluated antibody neutralization breadth against variants of concern using matched plasma from the Stanford COVID-19 Biobank. Matched plasma samples were from the same or closest blood draw (one donor’s closest plasma sample was +3 days and the remainder were from the same draw). All individuals were infected with ancestral SARS-CoV-2, equalizing our measurement of breadth against variants of concern. To stratify participants by breadth against variants of concern, we measured serum neutralization against Wu-1 (ancestral) and four variant SARS-CoV-2 pseudoviruses that emerged after patient samples were collected (alpha, beta, delta and original omicron B.1.1.529) in a HeLa cell line stably expressing ACE2 and TMPRSS2^31^ (Fig. 1a–d). We found that there was heterogeneity in both the neutralization titer against each variant and in the number of variants neutralized (Fig. 1b–d). The median 50% neutralization titer (NT50; the reciprocal of the highest dilution to obtain <50% infection) against Wu-1 was the highest with ~1 log of variation in NT50 across individuals. The median NT50 of the cohort was reduced compared with Wu-1 in alpha, beta, delta and omicron B.1.1.529; this was significant in beta, delta and omicron (Fig. 1b). As our cohort showed little neutralizing activity against original omicron, as has been previously reported^6^, we did not include variants that emerged later. We defined each participant’s breadth score as the number of variants neutralized; breadth scores ranged from 0 to 4 (Fig. 1c). All individuals with a breadth score of 4 neutralized Wu-1, alpha, beta and delta; this neutralization profile represents the maximum breadth observed in our cohort of ancestral infection. To compare optimal breadth with all other individuals, those with a breadth score of 4 were classified as broad neutralizers, whereas those that neutralized different combinations of 0–3 variants were classified as narrow neutralizers (Fig. 1c,d).

As it is possible that severity could drive breadth via immune activation, we validated that the breadth score did not correlate with the WHO severity score (Fig. 1e). The severity score was also well distributed over different variant neutralization combinations (Fig. 1d). Therefore, we can be confident that immunological signatures of breadth cannot be primarily attributed to severity, and, by including participants across the entire severity spectrum, we can analyze immune correlates of antibody breadth regardless of immunological signatures of disease severity. In addition, the number of days between a positive test and sample collection did not correlate with breadth scores; therefore, breadth cannot be primarily attributed to acute or convalescent sample identity (Fig. 1f). Breadth score and breadth groups were assigned to each cell. As shown in Uniform Manifold Approximation and Projection for Dimension Reduction (UMAP) projections of scRNA-seq and CyTOF data, major immune cell types are represented in both broad and narrow neutralizers, allowing us to thoroughly investigate systemic contributions to a productive immune response with regard to antibody breadth (Fig. 1g).

### NK cells in narrow neutralizers of SARS-CoV-2 highly express ISGs and markers of activation

**Fig. 2:**
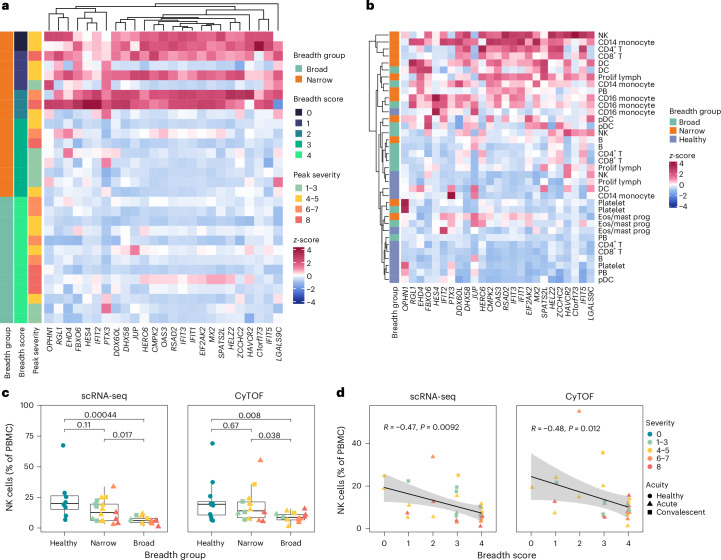
ISGs correlate with low breadth score and are highly expressed in NK cells. **a**, Heatmap of normalized expression of genes significantly correlated with low breadth score using DEseq2. Genes (columns) are ordered by hierarchical clustering; each row represents one donor. **b**, Heatmap of normalized expression of genes in **a** for each breadth group and cell type, ordered by hierarchical clustering for rows. **c**, Boxplots of proportion of NK cells in breadth groups from scRNA-seq and CyTOF datasets. Boxplots are drawn as median (center line), IQR (box) and 1.5× IQR (whiskers), colored by peak WHO severity score and shaped by acuity. *P* values calculated using two-sided Wilcoxon rank sum test with Bonferroni’s correction for multiple hypothesis testing. Each point represents one donor. **d**, Correlation between proportion of NK cells and breadth score in scRNA-seq and CyTOF datasets. Line of best fit and 95% confidence interval (CI) are shown. *P* values calculated using two-sided Spearman rank correlation. Each point represents one donor.

**Extended Data Fig. 2:**
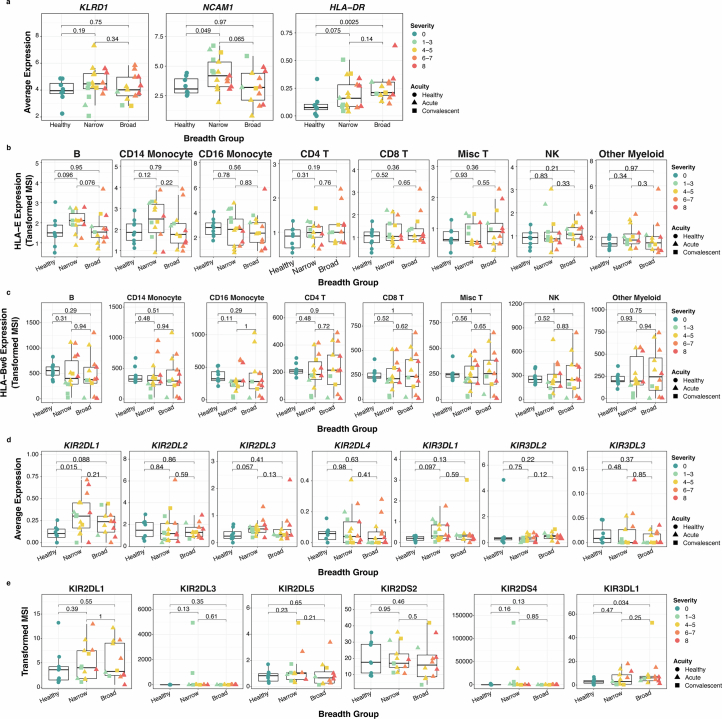
HLA and KIR Genotypes Across Breadth Groups. **a**. Boxplots quantifying average expression of remaining genes from Fig. 4b in scRNAseq dataset. (HLA-DR is the aggregated expression of HLA-DR genes *HLA-DRA* and *HLA-DRB1-5*.) **b**. Boxplots quantifying arcsinh-transformed MSI of HLA-E in each cell type from CyTOF dataset. **c**. Boxplots quantifying arcsinh-transformed MSI of HLA-bw6 in each cell type from CyTOF dataset. **d**. Boxplots quantifying expression of all KIR genes detected in scRNAseq dataset in NK cells. **e**. Boxplots quantifying arcsinh-transformed MSI of all KIRs included in CyTOF panel in NK cells. All boxplots are drawn as median (center line), IQR (box), and 1.5x IQR (whiskers), colored by peak WHO severity score, and shaped by acuity. P-values by two-sided Wilcoxon rank-sum test with Bonferroni’s correction for multiple hypothesis testing. Each point represents one donor.

To identify the most robust immunological signal that is correlated with breadth, we used pseudobulk whole PBMC samples from each patient to identify genes that significantly correlated with breadth score using DESeq2 (ref. ^32^). In whole PBMCs, we identified 25 genes whose expression significantly correlated with low breadth score, 24 of which were ISGs, verified by the interferome database including classical ISGs such as MX family, OAS family and IFI family genes^33^ (Fig. 2a). We next examined which cell types in narrow and broad breadth groups expressed these genes and found that NK cells from narrow neutralizers were among the highest expressors of the genes correlated with a low neutralization score (Fig. 2b). We also found NK cells to be one of only two cell types with differences in cell type proportion between broad and narrow neutralizers: broad neutralizers have significantly fewer NK cells when compared with narrow neutralizers and healthy controls (Fig. 2c). This trend further applied to breadth score, where NK cells as a proportion of PBMCs are inversely correlated with breadth score in both scRNA-seq and CyTOF modalities (Fig. 2d). Plasmacytoid dendritic cells (pDCs) (<1% of PBMCs in most participants) were significantly more abundant in narrow neutralizers in scRNA-seq data (Extended Data Fig. 2b,c). This perturbation of NK cells between breadth groups and breadth scores motivated us to further analyze the role of the NK cell compartment in modulating antibody breadth.

**Fig. 3:**
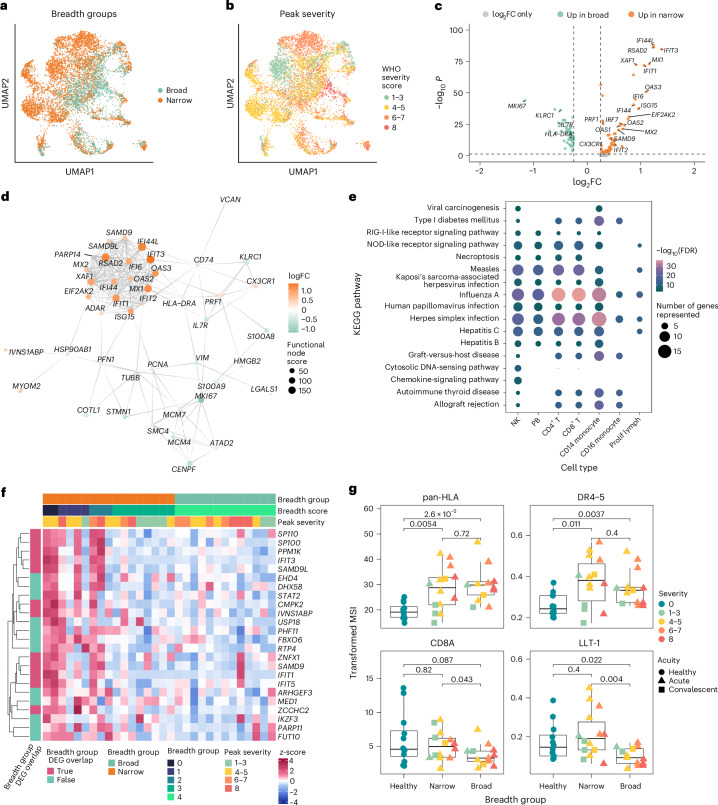
ISGs and abundant NK cells correlate with narrow neutralization breadth in COVID-19. **a**, UMAP of NK cells from scRNA-seq dataset colored by breadth group. **b**, UMAP of NK cells from scRNA-seq dataset colored by peak WHO severity score. **c**, Volcano plot of DEGs between broad and narrow neutralizers in NK cells from scRNA-seq dataset. *P* values calculated using two-sided Wilcoxon rank sum test with Bonferroni’s correction for multiple hypothesis testing. **d**, Protein–protein interaction graph depicting minimal significant interaction graph of NK cell DEGs (FDR <1 × 10^−6^). **e**, Dot plot of KEGG pathways significantly enriched in narrow neutralizer NK cells, showing their expression in each cell type. Plot colored by FDR and sized by number of genes present in KEGG pathway, with rows ordered by hierarchical clustering. **f**, Heatmap of normalized expression of NK cell genes significantly correlated with low breadth score using DEseq2. Each column represents one donor. **g**, Boxplots quantifying arcsinh-transformed mean signal intensity (MSI) of markers from CyTOF dataset in NK cells. Boxplots are drawn as median (center line), IQR (box) and 1.5× IQR (whiskers), colored by peak WHO severity score and shaped by acuity. *P* values calculated using two-sided Wilcoxon rank sum test with Bonferroni’s correction for multiple hypothesis testing. Each point represents one donor. Source data

In UMAP space, there was separation of NK cells by both severity and breadth groups (Fig. 3a,b). We identified differentially expressed genes (DEGs) in NK cells between broad and narrow breadth groups (log_2_fold change (FC) >0.25 and adjusted *P* value <0.05). NK cells from narrow neutralizers exhibit higher expression of multiple ISGs such as *IFI44L*, *RSAD2*, *XAF1*, *IFIT3*, *MX1*, *IFIT1* and so on as well as perforin (*PRF1*), a marker of NK cell cytotoxicity (Fig. 3c and Supplementary Table 2). NK cells from narrow neutralizers also upregulated *CX3CR1*, a marker of migration to lymphoid tissues where NK cells could influence antibody responses and that is associated with cytotoxic function^34^.

To visualize the interaction landscape and underlying regulators of DEGs in NK cells, we leveraged the Bionet package to find the highest-scoring subgraph (false discovery rate (FDR) <1 × 10^−6^) of DEGs from a background protein–protein interaction network derived from the human STRING database^35–37^. The minimal significant network of DEGs from NK cells revealed a hub of highly differentially expressed and interacting ISGs (*MX1*, *IFIT1*, *ISG15* and so on) in narrow neutralizers with high predicted functional node scores relative to other nodes (Fig. 3d). This confirms a high level of ISG-mediated activation in NK cells from narrow neutralizers. *CX3CR1* and perforin (*PRF1*) were also included in the minimally significant graph, highlighting both the migratory and cytotoxic potential of NK cells from narrow neutralizers.

Using DEGs upregulated in narrow neutralizers across all cell types, we performed pathway analysis to identify Kyoto Encyclopedia of Genes and Genomes (KEGG) pathways enriched in narrow neutralizers. NK cells from narrow neutralizers were enriched for multiple pathways related to viral infections and pathological inflammation. These pathways were also significantly enriched in multiple immune cell types such as T cells and classical (CD14) monocytes in narrow neutralizers (Fig. 3e). This indicates that narrow neutralizers have heightened inflammation and ISG expression across the immune system. We also repeated our pseudobulk correlation analysis using DESeq2 to find genes significantly correlated with breadth score in NK cells (Fig. 3f). We found that all genes significantly correlated with a low breadth score were ISGs (verified by interferome database)^33^; the majority overlapped with scRNA-seq DEGs from breadth groups (Fig. 3c,f). On the protein level, NK cells from all participants with COVID-19 exhibited upregulation of human leukocyte antigen (HLA) and death receptors (DR4–5); however, narrow neutralizers expressed significantly greater levels of CD8A and LLT-1 (markers of cytotoxicity and activation, respectively) when compared with broad neutralizers^38,39^ (Fig. 3g). This reveals specific activation markers present only in narrow neutralizer NK cells in the setting of other signals of disease-driven activation.

### NK cells are immature and proliferating in broad neutralizers of SARS-CoV-2

**Fig. 4:**
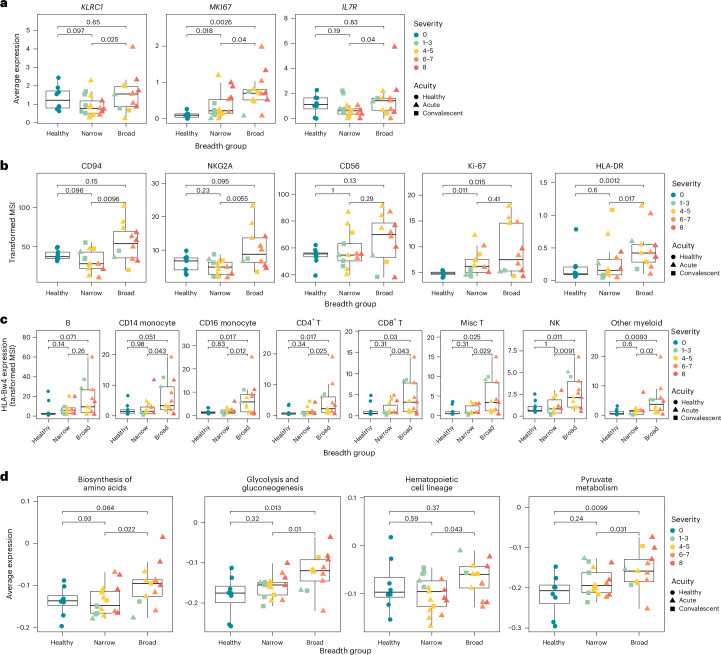
NK cells from broad neutralizers are immature, proliferative and enriched for normal processes. **a**, Boxplots quantifying expression of selected DEGs in NK cells from scRNA-seq dataset. **b**, Boxplots quantifying arcsinh-transformed MSI of selected markers in NK cells from CyTOF dataset. **c**, Boxplots quantifying arcsinh-transformed MSI of HLA-bw4 across cell types from CyTOF dataset. **d**, Boxplots quantifying average expression of KEGG modules in NK cells from scRNA-seq data. All boxplots are drawn as median (center line), IQR (box) and 1.5× IQR (whiskers), colored by peak WHO severity score and shaped by acuity. *P* values calculated using two-sided Wilcoxon rank sum test with Bonferroni’s correction for multiple hypothesis testing. Each point represents one donor.

Distinct from narrow neutralizers, in broad neutralizers, NK cells were significantly less abundant, but they express higher transcript levels of *MKI67* (Ki-67), *KLRC1* (NKG2A) and *IL7R* (CD127), indicating a proliferative, inhibitory and immature phenotype^40,41^ (Figs. 3c and 4a). Differential expression analysis indicates upregulation of some genes involved in immune activation such as *HLA-DRA* but no evidence of canonical ISG activation (Fig. 3c). This was substantiated on the protein level, where NK cells from broad neutralizers expressed significantly higher levels of NKG2A/CD94. CD56 expression was elevated in broad neutralizers, also supporting an immature phenotype (Fig. 4b). HLA-DR was upregulated on the protein but not the transcript level. Similarly, *KLRD1* (CD94) and *NCAM1* (CD56) were not differentially expressed in RNA but can have poor correlation between transcript and protein expression^42^ (Extended Data Fig. 2a). In addition, HLA-E, the ligand for NKG2A/CD94 heterodimer, is not differentially expressed on any cell type between broad and narrow neutralizers at the protein level^43^ (Extended Data Fig. 2b).

All cell types in broad neutralizers express higher levels of HLA-Bw4 (significant in all cell types except B cells), whereas expression of HLA-Bw6 is not different between broad and narrow neutralizers (Fig. 4c and Extended Data Fig. 2c). HLA-Bw4 and HLA-Bw6 represent mutually exclusive epitopes on class I HLA-B, and HLA-Bw4 but not HLA-Bw6 is directly recognized by NK cells and is correlated with protection from COVID-19 and control of human immunodeficiency virus^44^. This may support the partial contributions of genetics to productive antibody responses to SARS-CoV-2. A KIR3DL1 + HLA-Bw4+ genotype is also associated with protection from severe COVID-19 (ref. ^45^); however, neither KIR3DL1 nor any other KIR measured in CyTOF or detected in scRNA-seq was differentially expressed between any group (Extended Data Fig. 2d,e). Using genes significantly upregulated in NK cells from broad neutralizers, we performed pathway analysis using the KEGG pathway database^46^. We found upregulation of multiple pathways indicating normal cell functions and immaturity in NK cells from broad neutralizers (Fig. 4d and Supplementary Table 2).

### NK cell transcriptomic clusters enriched in narrow neutralizers express cytotoxic proteins and differentiation markers

**Fig. 5:**
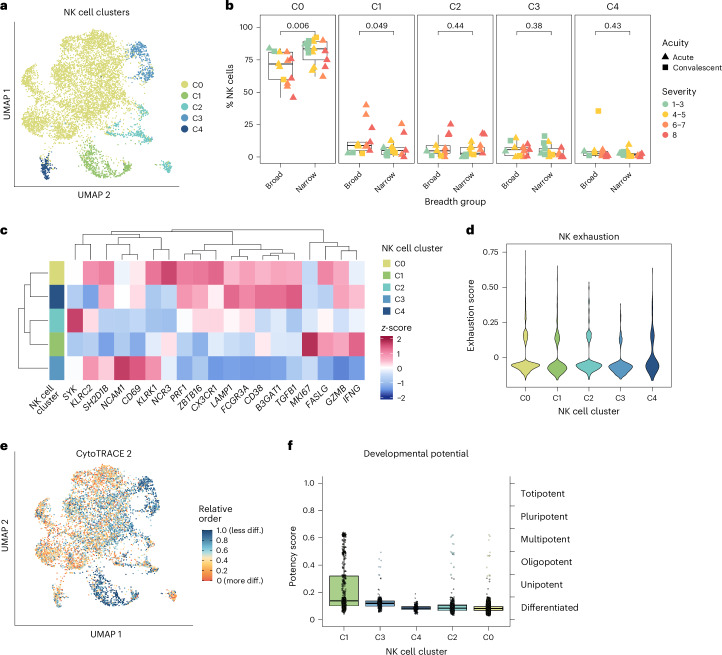
Mature NK cells are enriched in narrow neutralizers of SARS-CoV-2. **a**, UMAP of NK cells from scRNA-seq data colored by Seurat cluster. **b**, Boxplot NK cell cluster frequencies in each breadth group colored by peak WHO severity score and shaped by acuity. *P* value calculated using two-sided Wilcoxon rank sum test with Bonferroni’s correction for multiple hypothesis testing. Each point represents one donor. **c**, Heatmap of normalized gene expression of NK cell phenotypic and functional markers for each cluster. **d**, Violin plot of NK cell exhaustion (defined as average expression of *LAG3*, *PDCD1* and *HAVCR2*; Methods) for each cluster. **e**, UMAP of NK cells from scRNA-seq data colored by relative differentiation calculated by cytoTRACE2. **f**, Boxplots of potency score of each NK cell cluster calculated by cytoTRACE2 ordered by potency. All boxplots are drawn as median (center line), IQR (box) and 1.5× IQR (whiskers). Diff., differentiation.

To further investigate the phenotype of NK cells enriched in broad and narrow neutralizers, we applied unsupervised clustering to scRNA-seq data of all NK cells from participants with COVID-19 (Fig. 5a). We found five biologically relevant clusters, of which C0 was significantly enriched in narrow and C1 in broad neutralizers (Fig. 5b). NK cells in C0 are CD56^dim^CD16^high^ mature NK cells with higher co-expression of *B3GAT1* (CD57) and *KLRC2* (NKG2C) than in other clusters. C0 also expresses high transcript levels of *KLRK1* (NKG2D), *NCR3* (NKp30), *FASLG* (Fas-L), *LAMP1* (CD107a), *PRF1* (perforin) and *GZMB* (granzyme B), indicating cytotoxic potential, and had intermediate expression of *CD69* and high levels of *CD38*, demonstrating activation. Owing to the expression of *B3GAT1* and *KLRC2*, we investigated C0 and other clusters’ expression levels of receptors and transcription factors involved in the function of human cytomegalovirus (HCMV)-associated adaptive NK cells, which have been shown to lack cytotoxic function against autologous T cells^47^. Distinct from HCMV-associated adaptive NK cells, NK cells in C0 express intermediate levels of *SYK* and high levels of *FCGR3A* (CD16), *SH2D1B* (EAT-2) and *ZBTB16* (PZLF), all of which have low or absent expression in HCMV-associated adaptive NK cells. They also expressed the highest levels of *NCR3* (NKp30), which is lacking on HCMV-associated adaptive NK cells. Thus, the C0 cluster enriched in narrow neutralizers is not representative of HCMV-associated adaptive NK cells but appears to have a phenotype consistent with high cytotoxic function and maturity. NK cells in the C1 cluster (enriched in broad neutralizers) are CD56^dim^CD16^low^ but exhibit lower expression of activation markers *CD38* and *CD69* and cytotoxic markers *KLRK1*, *NCR3*, *LAMP1* and *PRF1*. However, the C1 cluster still highly expresses some cytotoxicity-related genes (*FASLG* and *GZMB)* and the cytokine *IFNG* (IFNγ). Thus, the cluster of NK cells enriched in broad neutralizers represents NK cells with diminished activation and more potential for cytokine secretion. The C1 cluster also expressed high levels of *MKI67* (Ki-67), further validating the increased proliferation of NK cells in broad neutralizers as compared with narrow neutralizers. Similar to HCMV-associated adaptive NK cells, C1 NK cells expressed low levels of *NCR3, FCGR3A*, *SYK*, *SH2D1B* and *ZBTB16*, while not expressing *B3GAT1* or *KLRC2*. C1 NK cells illustrate that lack of NKp30 along with low expression of SYK, EAT-2 and PZLF may prevent killing of autologous cells in nonadaptive NK cell populations. We did not observe a clear population in C0–C4 of bona fide HCMV-associated adaptive NK cells, potentially owing to the low prevalence of HCMV in Santa Clara county where these samples originated^48,49^. The remaining clusters, C2–C4, are not differentially expressed between broad and narrow neutralizers. C2 consists of CD56^dim^CD16^high^ NK cells expressing low levels of activation markers. C3 comprises CD56^bright^ NK cells expressing cytotoxic NK cell receptors but not markers of degranulation. NK cells in C4 are CD56^dim^CD16^high^ and express markers of degranulation but low levels of cytotoxic receptors (Fig. 5c).

In the setting of severe COVID-19, NK cells can become functionally exhausted while still expressing cytotoxic molecules^50–54^. Thus, we evaluated whether the highly activated NK cells in narrow neutralizers might be exhausted. We analyzed aggregated expression of canonical exhaustion markers (*LAG3*, *PDCD1* and *HAVCR2*) to generate an exhaustion score. Although all clusters exhibited some evidence of exhausted cells, the C0 cluster enriched in narrow neutralizers was not predominantly composed of cells expressing exhaustion genes (Fig. 5d). Thus, the expression of cytotoxic proteins observed in C0 is distinct from the exhaustion observed in severe COVID-19, in which NK cells do not retain intact cytotoxic function. Finally, we applied CytoTRACE2, a predictive method to evaluate absolute developmental potential in scRNA-seq data, to all NK cells from participants with COVID-19 (ref. ^55^) (Fig. 5e). We found that C1 contained the most immature cells with the highest developmental potential, while C0 was the most differentiated, consistent with their expression of CD57 and NKG2C and further supporting the observation that broad neutralizers are associated with immature NK cells and narrow neutralizers are associated with differentiated NK cells (Fig. 5f).

### Cell–cell interaction predictions indicate differences in cell signaling between broad and narrow neutralizers

**Fig. 6:**
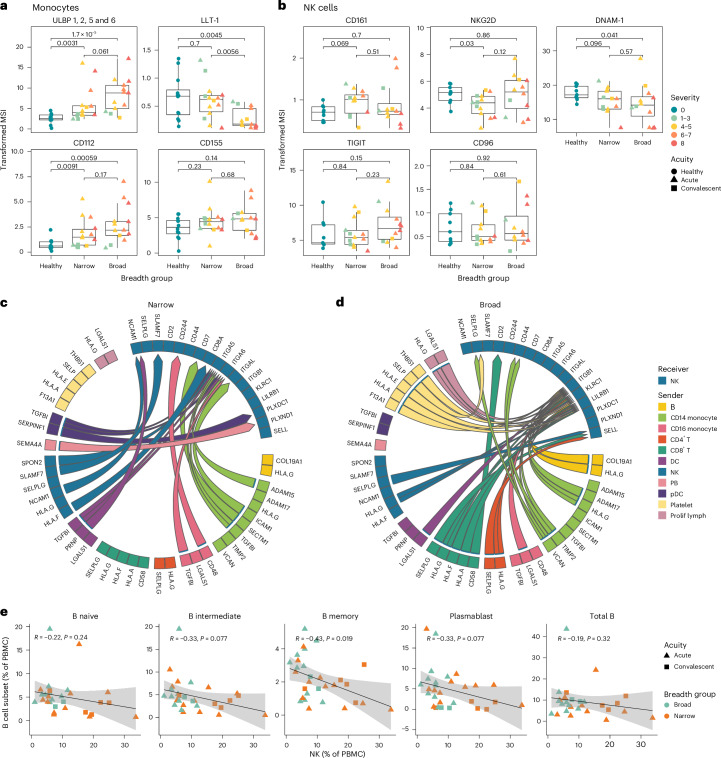
Cell-communication differences in broad and narrow neutralizers. **a**, Boxplots quantifying arcsinh-transformed MSI of NK cell ligands on monocytes in CyTOF data. **b**, Boxplots quantifying arcsinh-transformed MSI of cognate NK cell receptors in CyTOF data. All boxplots are drawn as median (center line), IQR (box) and 1.5× IQR (whiskers), colored by peak WHO severity score and shaped by acuity. *P* values calculated using two-sided Wilcoxon rank sum test with Bonferroni’s correction for multiple hypothesis testing. Each point represents one donor. **c**,**d**, Circos plot showing top 50 predicted cell–cell communication pairs sent to NK cells colored by sender cell in narrow (**c**) and broad (**d**) neutralizers. **e**, Correlation between B cell subset and NK cell frequency colored by breadth group and shaped by acuity. Line of best fit and 95% CI are shown. *P* values calculated using two-sided Spearman rank correlation. Each point represents one donor. Source data

Given that multiple immune cell types in narrow neutralizers demonstrated inflammatory phenotypes, we next investigated how cell–cell interactions may contribute to differential NK cell phenotypes in broad and narrow neutralizers. Our laboratory has previously found that monocytes contribute to NK activation and dysfunction in severe COVID-19 by interacting with NK cells both through direct receptor–ligand interaction and via cytokine secretion^56^. On the protein level, we found that monocytes in narrow neutralizers express significantly higher levels of LLT-1 compared with broad neutralizers (Fig. 6a). LLT-1 can activate NK cells and is downregulated in moderate and severe COVID-19 (ref. ^30^). LLT-1 binds CD161 on NK cells that is not differentially expressed^57,58^ (Fig. 6b). ULBPs 1, 2, 5 and 6—ligands for the NK cell activating receptor NKG2D—were elevated in monocytes from broad neutralizers, while CD112 and CD155 (ligands for DNAM-1, TIGIT and/or CD96 on NK cells) were not differentially expressed between breadth groups^59–62^ (Fig. 6a). Cognate NK cell receptors NKG2D, DNAM-1, TIGIT and CD96 were also not differentially expressed between breadth groups on NK cells (Fig. 6b).

**Extended Data Fig. 3:**
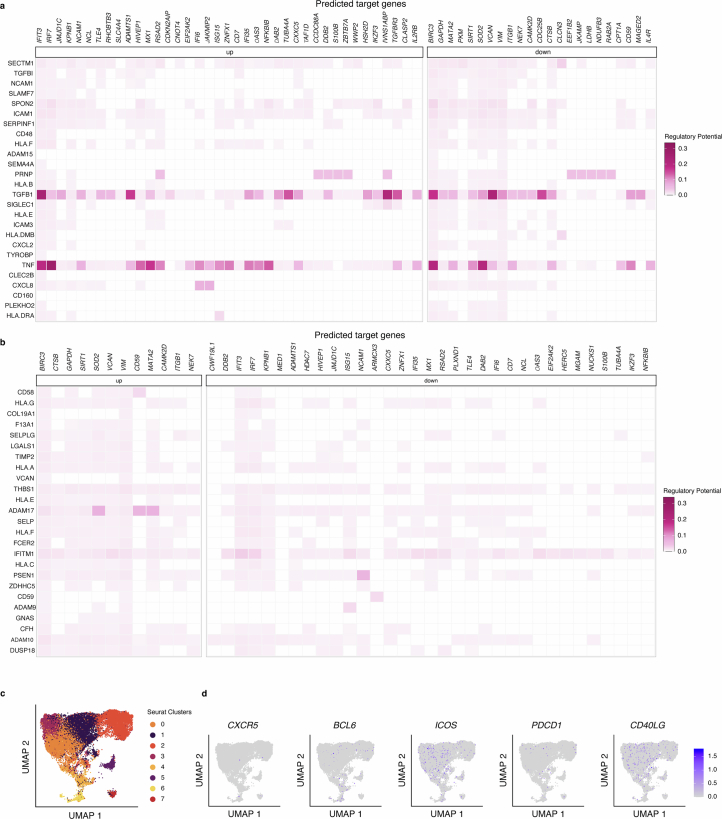
Predicted Target Gene Regulation in Cell-Cell Communication and Analysis of CD4 T Cell Clusters. **a**-**b**. Heatmap of regulatory potential of predicted downstream gene expression of the top 50 ligand-receptor pairs sent from all PBMC cell types to NK cells from narrow (a) and broad (b) breadth groups. **c**. UMAP of CD4 T cells in scRNA-seq data colored by seurat clusters. **d**. UMAP of CD4 T cells colored by expression of Tfh-cell-associated genes.

To predict cell–cell communication from scRNA-seq data, we used the Multinichenetr package to identify the top differentially expressed receptor–ligand interaction pairs between breadth groups. The MultiNicheNet method leverages the downstream signaling database NicheNet-v2 which integrates ligand–receptor, signaling and gene regulatory data into a network model that allows linkage of ligand–receptor interactions with active downstream signaling^63^ (Fig. 6c,d). There were distinct patterns of predicted cell–cell communication received by NK cells in broad and narrow neutralizers. In narrow neutralizers, multiple cell types (CD14 monocytes, CD16 monocytes, pDCs, platelets and dendritic cells (DCs)) were predicted to signal via *TGFBI* (TGFβ induced protein) through integrins (*ITGA5* and *ITGA6*), which inhibits adhesion and migration^64^. We also investigated predicted downstream signaling targets of both TGFβI and TGFβ in the top 50 predicted cell–cell communication pairs in narrow neutralizers. TGFβI (*TGFBI*) had low regulator potential to drive ISG expression, but TGFβ (*TGFB*, upstream of *TGFBI*) is predicted to drive expression of many ISGs including *MX1* and *ISG15* in narrow but not broad neutralizers (Extended Data Fig. 3a,b). TGFB and *IRF7* co-expression in the context of viral infection can drive type I interferon and ISG expression^65,66^. Here, *IRF7* was significantly upregulated in narrow neutralizers, which may work in conjunction with TGFβ signaling to drive interferon-stimulated gene expression in narrow neutralizers. Other sources of activation included *CD48* interaction with 2B4 (*CD244*)^67^ on CD16 monocytes and *CD7*-mediated activation by CD14 monocytes through *SECTM1* (ref. ^68^). NK cells themselves were also predicted to send auto-activation signals via *SLAMF7*–SLAMF7 (refs. ^69,70^), CD56–CD56 (*NCAM1*)^71^ and *CD8*–*HLA-F*^72^ interactions. There were also predicted interactions between *SPON2* and integrin alpha 5 (*ITGA5*) in NK cells from narrow neutralizers, which can inhibit migration^73^. In addition, signals involved in the inhibition of angiogenesis (*SEMA4A*-*PLXND1*^74^ and *SERPINF1*-*PLXDC1*^75^) were predicted from pDCs and plasmablasts (Fig. 6c).

CD4^+^ T cells, CD8^+^ T cells, proliferating lymphocytes, platelets and B cells were only predicted to communicate with NK cells in broad neutralizers. Here, signaling was dominated by inhibitory receptors; HLA-E, HLA-A, HLA-F and HLA-G were predicted to signal in multiple cell types through NKG2A (*KLRC1*) and/or LILRB1^40,43,76–80^. Receptor–ligand pairs involved in adhesion and lymphocyte homing were also predicted with signals from galectin 1 (*LGALS1*) from proliferating lymphocytes and CD16 monocytes; selectin P ligand (*SELPLG*) from NK cells, CD4^+^ T cells and CD8^+^ T cells; versican (*VCAN*) from monocytes; selectin P (*SELP*) from platelets and other signals through integrins across multiple cell types^81–84^. Some of the integrin-mediated signaling predicted in broad neutralizers are known to be inhibitory, such as *TIMP2* interaction with integrin beta 1 (*ITGB1*)^85^. Platelets are predicted to send inhibitory signals in broad neutralizers via selectin P (*SELP*), *HLA-A*, *HLA-E* and *THBS1*. Additional relevant signaling included immune synapse proteins *CD58* and *CD2* as well as *TIMP2*-*CD44* that can also be involved in migration and activation^86,87^ (Fig. 6d). Overall, these findings suggest that signals sent to NK cells in narrow neutralizers drive activation, including ISG expression as well as inhibition of migration and adhesion, and broad neutralizers are characterized by dominant inhibitory signals as well as positive regulation of migration and immune synapse formation.

Although predicted cell–cell communication between NK cells and T_FH_ is of particular interest, we re-clustered CD4^+^ T cells and did not identify T_FH_ in our scRNA-seq dataset. This is expected, as T_FH_ are extremely rare in blood^88,89^ and too few cells are evaluated by scRNA-seq for T_FH_ to be well represented (Extended Data Fig. 3c,d). However, we hypothesized that we could capture the indirect effect of NK cells on T_FH_ by analyzing the correlation between NK cell and B cell prevalence. The proportion of memory B cells had a significant negative correlation with the proportion of NK cells (Fig. 6e). The proportion of intermediate B cells and plasmablasts also exhibited a negative correlation with the prevalence of NK cells, and this trend approached significance. Total B cells and naive B cells did not exhibit this same correlation. This suggests that NK cell activity is correlated with diminished frequency of the B cell subsets that are critical for antibody production and durable antibody-based memory.

### Interferon α-activated NK cells exhibit enhanced killing and upregulate cytotoxic markers when cocultured with iT_FH_-like cells

**Extended Data Fig. 4:**
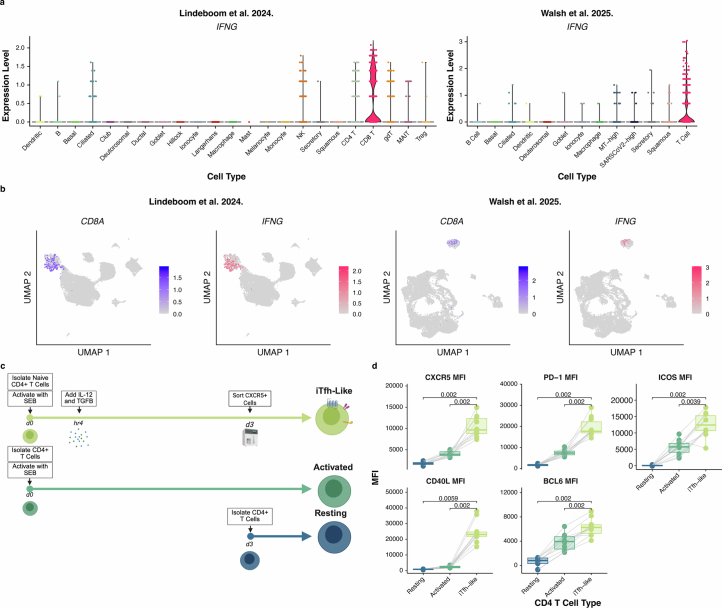
Tissue Expression of Interferons and *In Vitro* Differentiation of CD4 T cell types. **a**. Violin plot of single-cell level expression of *IFNG* in all cell types sequenced from nasopharyngeal swabs 7 days after challenge with ancestral SARS-CoV-2 (Lindeboom et al. Nature. 2024.) or PCR positive individuals during the ancestral wave (Walsh et al. Nat. Immunol. 2025). **b**. UMAP of *CD8A* and *IFNG* expression from scRNAseq data sets in a. **c**. Schematic of experimental timelines for *in vitro* differentiation of iTfh-like, activated, and resting CD4 T cells used in killing assays, co-cultures, and phenotypic profiling. **d**. Boxplots of MFI of Tfh markers in resting, activated, and iTfh-like CD4 T cells colored by CD4 T cell type. Boxplots are drawn as median (center line), IQR (box), and 1.5x IQR (whiskers). P-values by two-sided, paired Wilcoxon signed-rank test with Bonferroni’s correction for multiple hypothesis testing. Lines connect unique donors. (n = 10 across 4 experiments). Panel **c** created with BioRender.com. Source data

The predicted cell–cell communication in the peripheral immune system did not identify a source of interferons. We hypothesize that the source of interferon-mediated activation was in tissue. Using publicly available data of ancestral SARS-CoV-2 infection from nasopharyngeal swabs, we found that CD8^+^ T cells express *IFNG* in nasal tissue^90,91^ (Extended Data Fig. 4a,b). *IFNA* transcripts were not detected in scRNA-seq data across all datasets but are thought to be upstream of *IFNG* expression in T cells^92–94^. While we cannot look at differences in broad and narrow neutralizers in tissue expression of interferons, as no such data or samples exists, this shows that interferons are expressed in immune tissue during ancestral SARS-CoV-2 infection, which could disseminate to the blood, causing ISG expression observed in narrow neutralizers.

**Fig. 7:**
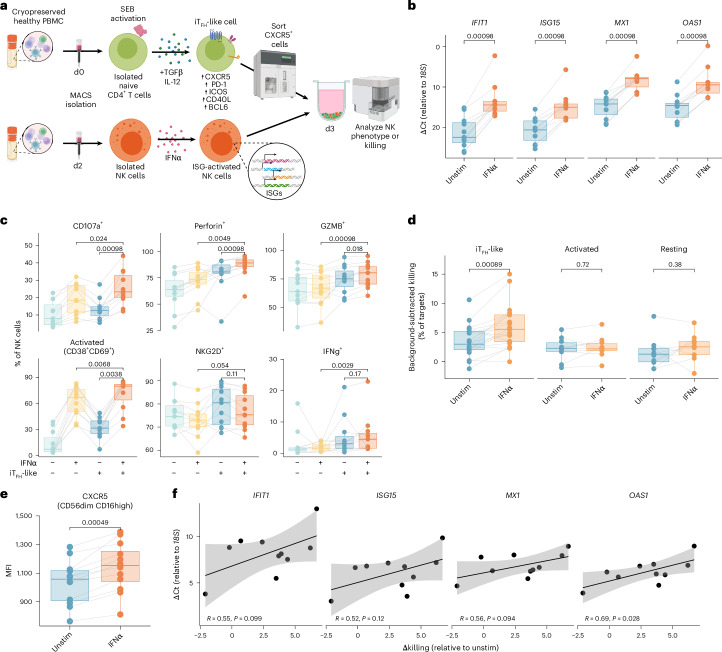
Interferon α-stimulated NK cells exhibit increased killing and markers of degranulation against iT_FH_-like cells. **a**, Experimental design of in vitro coculture experiments with healthy IFNα-activated NK cells cocultured with iT_FH_-like cells. **b**, Boxplots of ΔCt relative to 18S for *IFIT1*, *ISG15*, *MX1* and *OAS1* in NK cells from healthy donors unstimulated or activated with IFNα for 24 h. Each point represents the average of three technical replicates (*n* = 10 across three experiments). **c**, Boxplots of percentage of NK cells expressing cytotoxic and activation markers after 16-h coculture +/− iT_FH_-like cells (E:T = 1:5) and +/− NK activation with IFNα before coculture (*n* = 11 across three experiments). **d**, Boxplots of background-subtracted percentage of dead target cells in 3-h killing assay with iT_FH_-like, activated or resting CD4^+^ T target cells +/− NK activation with IFNα before killing assay (E:T = 5:1) (*n* = 18 across seven experiments for iT_FH_-like target cells, *n* = 10 across four experiments for activated and resting CD4 target cells). **e**, Boxplot of CXCR5 mean fluorescence intensity (MFI) on CD56^dim^ CD16^high^ NK cells +/− stimulation with IFNα for 24 h (*n* = 12 across two experiments). All boxplots are drawn as median (center line), IQR (box) and 1.5× IQR (whiskers). *P* values calculated using two-sided, paired Wilcoxon signed-rank test with Bonferroni’s correction for multiple hypothesis testing. Lines connect unique donors. **f**, Correlation between Δkilling (% killing IFNα pre-activated NK cells − % killing unstimulated NK cells) and ΔΔCt for expression of ISGs relative to unstimulated NK cells. Line of best fit and 95% CI are shown. *P* values calculated using Spearman rank correlation. Panel **a** created with BioRender.com. Source data

The activation and cytotoxic potential of NK cells in narrow neutralizers raises the hypothesis that NK cells may target T_FH_ in lymphoid tissue to limit antibody breadth. To directly assess the functionality of NK cells with narrow neutralizer phenotype against T_FH_, we developed an in vitro coculture system with healthy human donor NK cells and iT_FH_-like cells. Bona fide T_FH_ cells cannot be differentiated in vitro, but activation of primary, naive CD4^+^ T cells with TGFβ and interleukin (IL)-12 has been shown to induce high expression of CXCR5, ICOS and BCL6, which recapitulates key features of T_FH_ cells^95,96^. After isolation of naive CD4^+^ T cells from cryopreserved, healthy PBMCs, cells were activated with staphylococcal enterotoxin B (SEB; a T cell superantigen) for 4 h; then TGFβ and IL-12 were added to the culture for 72 h and CXCR5+ cells were sorted from this population. Sorted cells are referred to here as iT_FH_-like cells (Fig. 7a and Extended Data Fig. 4c). These iT_FH_-like cells were confirmed to express high levels of ICOS, PD-1, CD40L and BCL6 when compared with resting (freshly isolated) CD4^+^ T cells or CD4^+^ T cells activated for 72 h in the absence of cytokines, indicating a T_FH_-like phenotype (Extended Data Fig. 4d).

**Extended Data Fig. 5:**
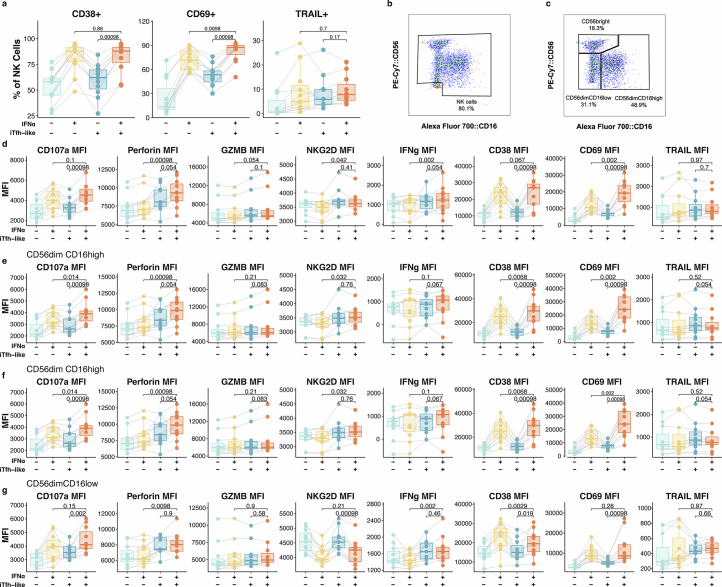
IFNα-Activated NK Cells Exhibit Increased Killing and Markers of Degranulation against iTfh-like Cells in all Subsets. **a**. Boxplots of percent of NK cells expressing cytotoxic and activation markers after 16-hr co-culture +/- iTfh-like cells (E:T = 1:5) and +/- NK activation with IFNα prior to co-culture. (n = 11 across 3 experiments). **b**. Representative flow plot of NK cells identified by CD16 and CD56 expression in flow cytometry data. **c**. Representative flow plot of gating to identify NK cell subsets using CD56 and CD16 expression. **d**. Boxplots of NK cell MFI for cytotoxic and activation markers after 16-hr co-culture +/- iTfh-like cells (E:T = 1:5) and +/- NK activation with IFNα prior to co-culture. (n = 11 across 3 experiments). **e**-**g**. Boxplots of MFI for cytotoxic and activation markers in CD56^dim^CD16^high^ (e), CD56^Bright^(f), and CD56^dim^CD16^low^ (g) NK cell subsets (gated in c) after co-culture described in d. (n = 11 across 3 experiments). All boxplots are drawn as median (center line), IQR (box), and 1.5x IQR (whiskers). P-values by two-sided, paired Wilcoxon signed-rank test with Bonferroni’s correction for multiple hypothesis testing. Lines connect unique donors. Source data

In order to mimic the high ISG expression in narrow neutralizer NK cells, we activated healthy, isolated NK cells with interferon α (IFNα) to induce ISG expression^51^. We found that in healthy donor NK cells, 24 h of activation with IFNα led to significantly greater expression (lower ΔCt relative to *18S*) of hallmark ISGs: *MX1*, *OAS1*, *ISG15* and *IFIT1* measured by reverse transcription quantitative PCR (qPCR) (Fig. 7b). We then investigated how NK cells pre-activated with IFNα responded to iT_FH_-like cells by coculturing them at an effector:target (E:T) ratio of 1:5 overnight (Fig. 7a). Following coculture, IFNα-activated NK cells expressed significantly more CD107a, perforin and granzyme B compared with unstimulated NK cells in coculture. Expression was also increased in coculture compared with IFNα-activated NK cells in the absence of target cells. This further indicates that NK cells respond specifically to iT_FH_-like target cells rather than IFNα activation alone. This trend was also observed in the percentage of activated (CD38+CD69+) NK cells. IFNα-activated NK cells in coculture also expressed more IFNγ than IFNα-activated cells cultured alone, but this difference was not significant between IFNα-activated and unstimulated NK cells in coculture. The proportion of NKG2D+ NK cells was slightly decreased in IFNα-activated cocultured NK cells, although this difference was not significant (Fig. 7c and Extended Data Fig. 5a). When we split NK cells by subtypes (that is CD56^dim^CD16^high^, CD56^dim^CD16^low^ and CD56^bright^), there were no major differences in responses to IFNα or iT_FH_-like target cells, with subsets following similar trends to bulk NK cells (Extended Data Fig. 5b–g).

**Extended Data Fig. 6:**
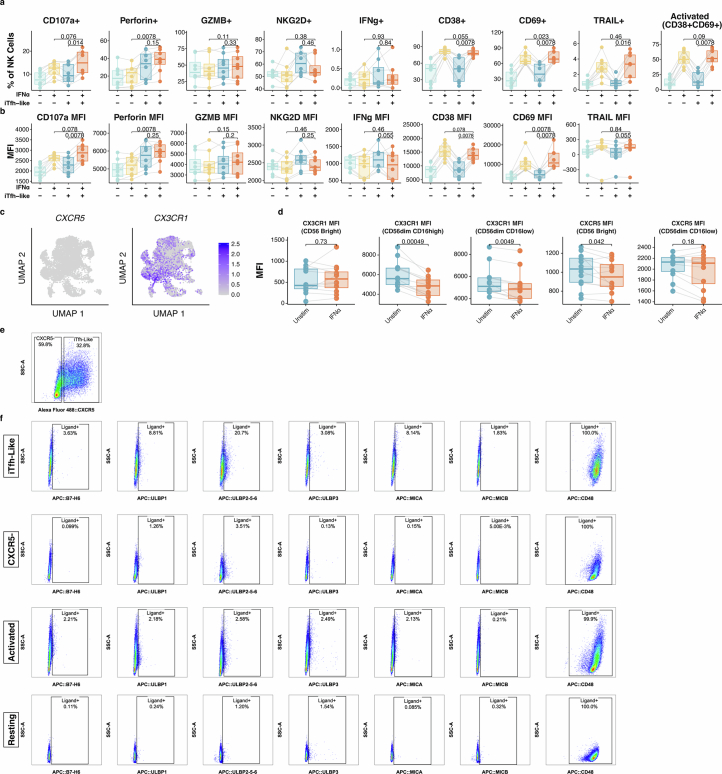
NK Cell Responses to Activated CD4 T cells, NK cell Expression of Migratory Receptors, and Expression of NK Cell Ligands on CD4 T Cell Types. **a**. Boxplots of percent of NK cells expressing cytotoxic and activation markers after 16-hr co-culture +/- activated CD4 T cells (E:T = 1:5) and +/- NK activation with IFNα prior to co-culture. (n = 8 across 2 experiments). **b**. Boxplots of NK cell MFI for cytotoxic and activation markers after 16-hr co-culture +/- inactivated CD4 T cells (E:T = 1:5) and +/- NK activation with IFNα prior to co-culture. (n = 8 across 2 experiments). **c**. UMAP of NK cells from COVID-19 subjects colored by *CXCR5* and *CX3CR1* expression. **d**. Boxplots of CX3CR1 and CXCR5 MFI in healthy donor NK +/- activation with IFNα. Graphs represent expression levels in NK cell subsets gated in Extended Data Fig. 5c. (n = 12 across 2 experiments). All boxplots are drawn as median (center line), IQR (box), and 1.5x IQR (whiskers). P-values by two-sided, paired Wilcoxon signed-rank test with Bonferroni’s correction for multiple hypothesis testing. Lines connect unique donors. **e**. Representative flow plot depicting gating of iTfh-Like and CXCR5- cells from CD4 + , live, single cells. **f**. Representative flow plots depicting NK cell ligand expression on iTfh-like, CXCR5-, activated, and resting CD4 T cells. Source data

We also performed killing assays with IFNα-activated NK cells against iT_FH_-like cells at an E:T ratio of 5:1 for 3 h. We found that IFNα-activated NK cells killed significantly more iT_FH_-like cells than unstimulated NK cells (median difference 2.6%) (Fig. 7d). While NK cells have been reported to kill activated CD4^+^ T cells, we determined that interferon-driven killing is iT_FH_-like specific because IFNα-activated NK cells did not kill significantly more activated or resting CD4^+^ T cells. We further investigated the specificity of interferon-driven response to iT_FH_-like cells by repeating coculture experiments overnight at E:T 1:5 with activated CD4^+^ T cells. We similarly observed that NK cell functional responses to activated CD4^+^ T cells were not IFNα dependent; for example, some markers such as perforin were upregulated by both unstimulated and IFNα-stimulated NK cells in response to activated CD4^+^ T cells and other markers such as CD38 were upregulated in response to IFNα but in both target cell and NK-only wells (Extended Data Fig. 6a,b). This shows that the IFNα-driven NK cell functional and cytotoxic response is specific to iT_FH_-like target cells and not target cell activation.

We also found evidence that IFNα activation and ISG expression may increase NK cell migratory potential to lymphoid tissues where T_FH_ reside. In CD56^dim^CD16^high^ cytotoxic NK cells, treatment with IFNα increased CXCR5 but not CX3CR1 expression, which could permit NK migration to lymph nodes (Fig. 7e). This in vitro IFNα activation did not recapitulate the higher expression of CX3CR1 in narrow neutralizers, possibly because in vivo activation signals were not fully captured in our system (Extended Data Fig. 6c,d). CXCR5 expression decreased in CD56^bright^ NK cells after IFNα activation in vitro. This subset is the most immature, similar to the NK cell phenotype in broad neutralizers, indicating that the NK cell phenotype enriched in broad neutralizers has lower migratory potential (Extended Data Fig. 6d). Finally, we found that the magnitude of upregulation of ISGs after IFNα activation (the absolute value of the difference in ΔCt values between IFNα-activated and unstimulated NK cells) correlated with the difference in killing of iT_FH_-like cells between IFNα-activated and unstimulated NK cells (ΔKilling), with greater cytotoxicity associated with greater increase in ISG gene expression. This correlation was only statistically significant for *OAS1*, and other genes approached significance (Fig. 7e). Overall, these data suggest that NK cells with narrow neutralizer phenotype have the capacity to specifically target autologous T_FH_ driven by ISG expression.

### NKG2D and NKp30 contribute to NK cell killing of iT_FH_-like cells

**Fig. 8:**
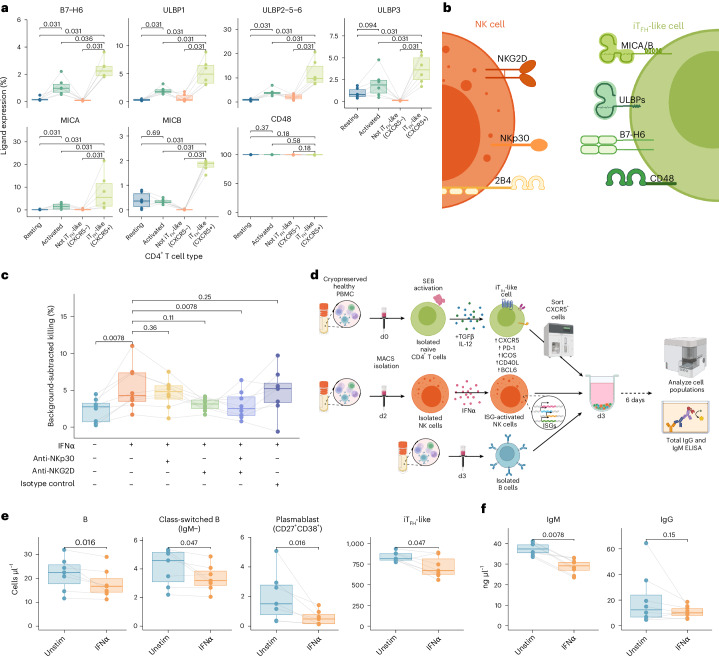
IFNα-activated NK cells target iT_FH_-like cells via NKp30 and NKG2D and limit B cell and antibody responses. **a**, Boxplots of percentage cells expressing NK ligands on iT_FH_-like (CXCR5+), CXCR5−, activated and resting CD4^+^ T cells. **b**, Diagram of NK cell ligands profiled on iT_FH_-like cells and their cognate NK cell receptors. **c**, Boxplots of background-subtracted percentage of dead target cells in 3-h killing assay +/− NKp30- and/or NKG2D-blocking antibodies with iT_FH_-like target cells +/− NK activation with IFNα before killing assay (E:T = 5:1) (*n* = 8 across two experiments). **d**, Experimental design of in vitro coculture system with healthy IFNα-activated NK cells, iT_FH_-like cells and B cells with analysis modalities. **e**, Boxplots quantifying cell numbers after 6 days of coculture of iT_FH_-like and B cells with NK cells +/− stimulation with IFNα (*n* = 8 across two experiments). **f**, Boxplots quantifying immunoglobulin concentration in cell culture supernatants after 6 days of coculture of iT_FH_-like and B cells with NK cells +/− stimulation with IFNα (*n* = 8 across two experiments). All boxplots are drawn as median (center line), IQR (box) and 1.5× IQR (whiskers). *P* values calculated using two-sided, paired Wilcoxon signed-rank test with Bonferroni’s correction for multiple hypothesis testing. Lines connect unique donors. Panels **b** and **d** created with BioRender.com. Source data

To understand the mechanism of NK cell targeting of iT_FH_-like cells, we profiled NK cell ligand expression on iT_FH_-like cells compared with resting and activated CD4^+^ T cells. Ligand expression was also assessed on CXCR5+ (iT_FH_-Like) and CXCR5− (not iT_FH_-like) populations derived from the unsorted pool of differentiated naive CD4^+^ T cells (Extended Data Fig. 6e). We quantified expression of B7-H6, ULBP1, ULBP2-5-6, ULBP3, MICA, MICB and CD48, ligands of activating receptors NKG2D, NKp30 and 2B4 (refs. ^59,60,97–99^) (Fig. 8a and Extended Data Fig. 6f). We found increased expression of B7-H6, ULBP1, ULBP2-5-6, ULBP3, MICA and MICB on iT_FH_-like cells compared with activated and resting cells. Although activation increased the expression of most ligands on CD4^+^ T cells compared with resting cells, iT_FH_-like cells showed even higher levels of B7-H6, ULBPs and MICA/B than those seen in activated CD4^+^ T cells or CXCR5− cells. CD48 was highly expressed on all CD4^+^ T cell types (Fig. 8a). To determine if NKp30 (binds B7-H6) or NKG2D (binds ULBPs and MICA/B) contribute to killing of iT_FH_-like cells, we blocked these receptors during killing assays against iT_FH_-Like cells (Fig. 8b). Blocking NKG2D or NKp30 alone did not significantly reduce IFNα-driven killing of iT_FH_-like cells. However, when we blocked both NKG2D and NKp30, IFNα-activated NK cells killed significantly less iT_FH_-like cells, comparable to the level of killing of unstimulated NK cells (Fig. 8c). This suggests that both NKG2D and NKp30 play a role in interferon-driven NK cell killing of T_FH_ and suppression of antibody responses. Notably, NK cell transcriptomic clusters enriched in narrow neutralizers expressed higher levels of NKG2D and NKp30, and IFNα-activated NK cells downregulated NKG2D in culture with iT_FH_-like cells, which can indicate use of this receptor that is internalized after ligand engagement^100^, further supporting a role for these activating receptors in NK-cell-mediated suppression of T_FH_ in our cohort (Figs. 5c and 7c).

### NK cell restriction of iT_FH_-like cells directly suppresses B cell responses in in vitro cocultures

Finally, to determine whether interferon-driven activation of NK cells can modulate antibody responses and B cell function via restriction of T_FH_ cells, we used an in vitro coculture system with NK, B and iT_FH_-like cells. Unstimulated or IFNα-activated NK cells were incubated with iT_FH_-like cells for 1 h at an E:T of 5:1 before addition of autologous B cells. B, iT_FH_-like and NK cells were cocultured for 6 days at a ratio of 2:1:5, and cell counts and supernatant antibody levels were evaluated using counting beads and enzyme-linked immunosorbent assay (ELISA), respectively (Fig. 8d). IFNα-activated NK cells suppressed survival and proliferation of iT_FH_-like cells in this long-term coculture system. This confirms that low-level differences in cytotoxicity against iT_FH_-like cells in short-term killing assays are amplified in long-term cocultures. After 6 days, there were also significantly fewer B cells surviving in coculture with IFNα-activated NK cells than with unstimulated NK cells. Moreover, the numbers of class-switched (IgM−) B cells and plasmablasts (CD38+CD27+) were also significantly reduced in coculture with IFNα-activated NK cells (Fig. 8e). The lower level of B cell survival and activation impacted antibody responses and secretion, with reduced levels of IgG and a significantly lower concentration of IgM in the supernatants from cocultures with IFNα-activated NK cells (Fig. 8f). Here, we confirm that direct effects of NK cell activity on iT_FH_-like cells impact specific B cell subsets such as plasmablasts (validates Fig. 6e) and also affect antibody production in vitro.

## Discussion

**Extended Data Fig. 7:**
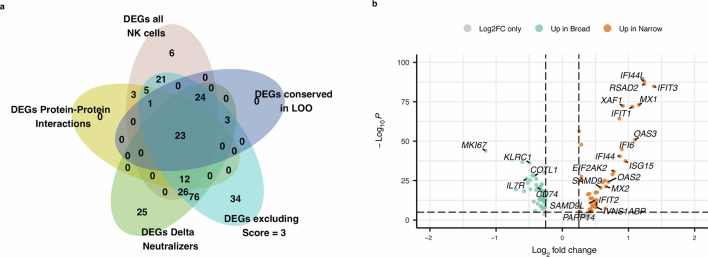
Differences in NK Cells are Robust to Breadth Group Definitions. **a**. Venn diagram of genes overlapping between different breadth group DEGs (See methods). **b**. Volcano plot of all NK cell DEGs from broad and narrow individuals with 23 conserved genes between all breadth group methods labeled. P-value by two-sided Wilcoxon rank-sum test with Bonferroni’s correction for multiple hypothesis testing.

The COVID-19 pandemic has highlighted the critical need to understand immunological determinants of antibody breadth to formulate actionable strategies to elicit broad protection with vaccines. Although NK cell activation has been repeatedly highlighted as a correlate of poor response to vaccines^10,11,18^, there is a dearth of research to elucidate the mechanism of human NK cell regulation of T_FH_ and antibody responses, particularly in the context of acute infection. To confidently investigate immunological correlates of antibody breadth, we classified individuals in our unique cohort of primary infection with ancestral SARS-CoV-2 into broad and narrow neutralizers on the basis of a biologically meaningful upper limit of breadth (breadth score of 4). Alternative breadth-group methods yielded highly conserved DEGs (Extended Data Fig. 7a,b; Methods). Together, these analyses establish robust breadth groups for uncovering differences between broad and narrow neutralizers. We leveraged multiomic approaches and our unique cohort in combination with in vitro functional validation to uncover that type I interferon activation and greater proportion of NK cells is correlated with narrow antibody response, whereas features of immaturity and inhibitory signaling in NK cells are correlated with broad antibody responses. We further demonstrate that interferon-driven activation of NK cells increases their response to and killing of autologous iT_FH_-like cells via NKG2D and NKp30, which directly impacts B cell responses, our hypothesized mechanism of NK cell regulation of antibody breadth.

ISGs are expressed in response to interferons such as IFNα and IFNγ and have critical antiviral functions^101^. In COVID-19, interferon signaling and its role in pathology or viral clearance has remained controversial, with early interferon signaling thought to promote viral clearance and persistent interferon signaling leading to greater disease severity^102–104^. Beyond its association with severity, we found an alternate role for interferon-mediated activation in NK cells: we showed, through multiple overlapping methods, that NK cells in narrow neutralizers show profound type I interferon activation. The abundant NK cells from narrow neutralizers exhibited significant upregulation of ISGs. In fact, 81/86 genes that were upregulated in narrow neutralizers were ISGs according to the interferome database^33^. NK cell clusters enriched in narrow neutralizers expressed multiple cytotoxic proteins and were the most differentiated of the NK cell clusters in our dataset. These NK cells also had migratory potential to traffic to lymphoid tissues owing to their expression of CX3CR1, which is a chemokine receptor that also defines highly cytotoxic, mature NK cells^34^. Overall, our data link ISG-mediated activation with increased cytotoxic potential of NK cells against T_FH_.

To further investigate the role of ISG activation in NK cells, we conducted in vitro experiments that demonstrated enhanced cytotoxic activity of IFNα-activated NK cells against autologous iT_FH_-like cells. While the difference in killing by IFNα-activated and resting NK cells is relatively small (median difference of 2.6%), this is consistent with a level of killing that could drastically affect antibody responses, where few T_FH_ are responsible for amplifying antigen-specific antibody responses. T_FH_ are the limiting factor in germinal center reactions, and intervention at this point in the immune response is greatly amplified over the course of viral infection^105^. The level of iT_FH_-like killing in IFNα-activated NK cells was correlated with ISG upregulation, supporting a proposed mechanism where ISG-mediated activation leads to NK-cell-mediated killing of autologous cells. In addition, killing of iT_FH_-like cells was specific, with no difference in killing against activated or resting CD4^+^ T cells.

Importantly, we identified specific activating ligands upregulated on iT_FH_-like cells that sensitize them to NK-cell-mediated killing via NKG2D and NKp30. Combined NKG2D and NKp30 blockade abrogated killing, reducing cytotoxicity to baseline. This receptor redundancy indicates that NK cell killing of iT_FH_-like cells is multifactorial, with both NKG2D and NKp30 engagement required for maximum cytotoxicity. These results provide mechanistic insight into how ISG-expressing NK cells selectively target T_FH_, highlighting an additional layer of regulation linking innate immune activation with poor breadth. Furthermore, in long-term cocultures, IFNα-activated NK cells reduced B cell survival, class switching, plasmablasts and antibody secretion. These findings reveal that ISG-expressing NK cells can functionally limit antibody titers through restriction of T_FH_, providing a mechanistic link between NK phenotype and antibody breadth observed in our cohort. Lastly, we observed that CD56^dim^CD16^high^ NK cells upregulated CXCR5 after in vitro stimulation with IFNα, aligning with reports of CXCR5 + NK cells in lymph node tissues^106,107^. While we did not see upregulation of CX3CR1 in vitro, a feature of NK cells from narrow neutralizers, it is possible that other inflammatory signals that overlap with ISGs cause expression of this receptor. Notably, we did not detect *CXCR5* transcripts in NK cells from our COVID-19 cohort, limiting our ability to directly compare the in vitro and in vivo migratory phenotype. Nevertheless, these findings suggest that ISG-driven migratory reprogramming of NK cells could contribute to their ability to access T cell zones and influence T_FH_ dynamics during acute infection via CXCR5 and/or CX3CR1.

In broad neutralizers, NK cells exhibited evidence of immaturity and proliferation with no ISG activation. NKG2A (marker of immaturity), which dimerizes with CD94 to send inhibitory signals, has been identified to play a role in multiple aspects of the productive immune response to SARS-CoV-2. SARS-CoV-2 viral peptides stabilize HLA-E (NKG2A/CD94 ligand) and prevent binding to NKG2A which ‘unleashes’ NKG2A + NK cells to eliminate SARS-CoV-2-infected target cells^108^. Thus, while NKG2A could be restricting NK cell killing of T_FH_ or regulation of adaptive immunity, these NKG2A+ cells could be unrestricted in killing of virally infected cells, leading to clearance of the virus. Furthermore, NKG2A was also identified as a predictor of response to the Moderna mRNA vaccine against SARS-CoV-2, indicating the clinical relevance of this trend^109^. HLA-Bw4, but not HLA-Bw6 expression levels, was also elevated in broad neutralizers. HLA-Bw4 has been associated with resistance to acquired immunodeficiency syndrome and clearance of SARS-CoV-2 (refs. ^44,45^). We further suggest that HLA-Bw4 may be associated with more productive antibody responses.

Our study identifies an association between NK cell responses and antibody breadth in acute human viral infection, although there are several limitations. Though we show that ISG expression in NK cells drives killing of autologous iT_FH_-like cells and suppresses humoral responses in vitro, further work is needed to directly observe NK–T_FH_–B cell interactions in human lymphoid tissue to confirm this model. Here, we were unable to directly profile T_FH_ owing to low cell numbers in scRNA-seq, lack of tissue samples, and CyTOF panel design. As a result, our mechanistic model relies on correlative effects on B cells in patient data and in vitro experiments with iT_FH_-like cells, which, although validated in their ability to support B cell responses, may not fully recapitulate in vivo T_FH_ biology. In addition, this study is limited to a single discovery cohort, and we lack an independent validation cohort to confirm transcriptomic associations with breadth owing to a lack of publicly available matched scRNA-seq and breadth data from the ancestral wave (March to October 2020). Future studies incorporating longitudinal sampling, lymphoid tissue or in vivo models will be critical for validating the mechanisms proposed here. Despite these limitations, the convergence of transcriptomic, proteomic and functional data in our study provides a compelling framework for future investigation of how NK cells modulate humoral immunity. We were also unable to sequence the B cell receptor repertoire in our cohort owing to sample availability; therefore, specific effects of NK cell activity on somatic hypermutation, antigen specificity or binding strength remain unknown. However, the identification of NK cell phenotypes associated with broad neutralization, including against unknown variants, represents actionable insight. This work provides a critical foundation for the rational design of vaccines aimed at maximizing breadth and cross-protection, regardless of specific mutational patterns induced.

Here, we used a systems immunology approach to identify an interferon-mediated mechanism driving NK cell activation and killing of autologous T_FH_ cells that may be the link between NK cells, poor vaccine response and limited antibody breadth. We additionally identify features of immaturity and inhibitory signaling in broad neutralizers of SARS-CoV-2, including NKG2A. Understanding these positive and negative correlates of breadth is critical to developing durable, broad-spectrum vaccines against COVID-19 and other emerging pathogens. Although the first vaccine against ancestral SARS-CoV-2 entered clinical trials only 128 days after the COVID-19 global pandemic was declared, efficacy estimates dropped as low as 9% against omicron B.1.1.529 (refs. ^110,111^). Similarly, passive antibody therapies initially showed efficacy but were rendered largely ineffective by the emergence of omicron and subsequent variants, highlighting the critical need to understand antibody breadth to anticipate and counter future variants^112^. Understanding broad antibody responses against novel variants will allow scientists to formulate actionable strategies to elicit broad protection with vaccines against emerging viruses or persistent viral challenges. In the future, we can use this understanding of the acute immune response against SARS-CoV-2 that leads to broad and narrow neutralization to select and design adjuvant–immunogen combinations that minimize ISG activation, specifically in NK cells, and maximize the potential of NKG2A + NK cells both against virally infected cells and in driving favorable antibody responses.

## Methods

### Sample collection

Samples were obtained from the Stanford COVID-19 Biobank with written informed consent under protocols approved by the Stanford University Institutional Review Board.

### Pseudotyped virus production

SARS-CoV-2-variant spike-pseudotyped lentiviral particles were produced in 293 T cells (ATCC) using Fugene transfection reagent 6 (Promega; E2691). Two million cells were seeded in D10 (DMEM (Life Technologies; 11885-092) + 10% FBS, 1% ʟ-glutamine, 1% Penicillin–Streptomycin–Amphotericin B, 10 mM HEPES) in T75 flasks 24 h before transfection. A three-plasmid system was used for viral production, as described by Ou et al. in 2020 (refs. ^113,114^). The spike vector contained the 21-amino-acid-truncated form of the SARS-CoV-2 sequence from the Wu-1 strain of SARS-COV2 (Wu-1 sequence ID no. BCN86353.1; alpha sequence ID no. QXN08428.1; beta sequence ID no. QUT64557.1; delta sequence ID no. QWS06686.1 with additional V70F and A222V mutations; and omicron sequence ID no. UFO69279.1). In a final volume of 1 ml, 1 μg Lenti backbone, 1.28 μg psPAX2 and 0.34 μg spike plasmid were mixed with serum-free DMEM. 100 μl fugene6 was added dropwise and incubated at room temperature for 15 min to form transfection complexes. The 1 ml transfection mix was then added dropwise to plated 293Ts. Culture medium was removed and replaced 24 h after transfection. Viral supernatants were collected 72 h after transfection by 0.45 μM filtration. Viral stocks were aliquoted and stored at −80 °C until further use (no less than 48 h).

### Pseudovirus neutralization assay and breadth score

Target cells were from the HeLa cell line stably overexpressing ACE2 and TMPRSS2, produced as described by Rogers et al. in 2020 (ref. ^113^). HeLa-ACE2-TMPRSS2 cells were thawed in D10 and passaged at least twice before use. Patient sera were inactivated at 56 °C for 1 h before use. Sera diluted 1:50 were filtered through a 0.22-μM spinX filter (Corning; 8160). Serial dilutions (1:2) of sera up to 1:1,600 was performed in 96-well black-walled, clear bottom plates (ThermoFisher; 165305) for a 25 μl final volume. A total of 25 μl pseudovirus was added at a titer such that virus-only wells would achieve a luminescence of ~100,000 RLU on a Promega Glomax plate reader (Promega; GM3000). Virus–serum mixtures were incubated for 1 h at 37 °C. HeLa-ACE2-TMPRSS2 cells were collected using trypLE (Gibco; 12604021). After incubation, 10,000 cells (50 μl) per well were added 48 h later, all media were removed from the plate by vacuum aspiration and cells were lysed by addition of 50 μl of britelite plus assay readout solution (Revvity; 6066766) and 50 μl 1× phosphate-buffered saline (PBS). Luminescence was measured using Promega Glomax after 30 s of shaking. Background luminescence (cell-only wells) was averaged and subtracted from all wells. Percentage neutralization was calculated by comparing with 100% infection (virus-only wells). The reciprocal of the highest dilution to obtain <50% infection was used to calculate the average NT50. The number of average NT50s above 100 (limit of detection) was used as breadth score.

### Processing of scRNA-seq

PBMC and whole blood samples were collected and processed in seqwell-based scRNA-seq as described by Wilk et al. in 2021 (ref. ^30^). The R package Seurat (V4.4.0)^42^ was used to subset participants included in this study and to remove neutrophils and developing neutrophils, as they were not preset for each participant. Subsetted data were rescaled and transformed using the SCTransform() function, and linear regression was performed to remove unwanted variation owing to cell quality. Principal component (PC) analysis was performed using the 3,000 most highly variable genes, and the first 50 PCs were used to perform UMAP to embed the dataset into two dimensions. Next, the first 50 PCs were used to construct a shared nearest neighbor graph (FindNeighbors()). Cell types identified by Wilk et al.^30^ were verified using known lineage markers. DEGs for each cell type were identified using Seurat’s FindMarkers() with the Wilcoxon rank sum test, log_2_FC > 0.25 and adjusted *P* value <0.05 (Supplementary Table 2).

### DESeq2 correlation analysis

Raw counts were subsetted by removing cells with <300 reads, >4,000 unique reads, >10,000 total reads, >15% mitochondrial reads, <1% mitochondrial reads, >7% ribosomal reads and >50% ribosomal RNA. Raw pseudobulk count matrices were constructed using the AggregateExpression() function in Seurat. Using DESeq2 (V1.38.3), count matrices were used to make a DESeq object (DESeqDataSetFromMatrix()) with design = ~Score. Genes that correlate with breadth score as a numerical variable were identified using an adjusted *P* value <0.05 (ref. ^32^).

### KEGG pathway analysis

Stringdb package (V2.10.1) was used to calculate gene set enrichment of DEGs from the KEGG pathway database^46,115^. All genes measured in scRNA-seq were used to set background using stringdb$set_background(). Enrichment was calculated using string_db$get_enrichment() hypergeometric test with Benjamini–Hochberg multiple hypothesis testing correction.

### Gene module scoring

The Seurat function AddModuleScore() was used to score single cells by expression of a list of genes. This function calculates a module score by comparing the expression level of an individual query gene with other randomly selected control genes expressed at similar levels to the query genes and is therefore robust to scoring modules containing both lowly and highly expressed genes, as well as to scoring cells with different sequencing depths.

### Bionet

A protein–protein interaction network of the 2,000 most DEGs in NK cells was used as the background interaction graph constructed using STRINGdb. Genes were identified using FindVariableFeatures(), with selection.method = vst and n.features = 2,000. The STRINGdb background graph is available in our GitHub repository (Code availability section). The Bionet R package (V1.58.0) to find the highest-scoring subgraph of DEGs (scoreNodes(FDR 1 × 10^−6^))^36,37^. The minimal significant network was calculated using runFastHeiz(), and nodes were colored by logFC from differential expression analysis.

### Analysis of breadth-group reproducibility

Seurat’s FindMarkers() using a two-sided Wilcoxon rank sum test, log_2_FC > 0.25 and adjusted *P* value <0.05 was used for different breadth-group definitions to identify DEGs conserved between different methods of identifying broad and narrow neutralizers (Extended Data Fig. 7). Narrow neutralizers as defined by a breadth score <4 were compared with broad neutralizers as described in the manuscript (DEGs all NK cells). Delta neutralizers were compared with delta nonneutralizers (DEGs delta neutralizers). Broad neutralizers (breadth score of 4) were compared with narrow neutralizers excluding individuals with a breadth score of 3 (DEGs excluding score = 3). Finally, each individual was excluded from DEG analysis between breadth groups in a leave one out (LOO) framework, and conserved DEGs from all iterations were reported (DEGs conserved in LOO). These DEG lists were also compared with the DEGs highlighted in the Bionet minimally significant graph, which represents protein–protein interactions among DEGs. Overlap in these DEG definitions were visualized using the VennDiagram (V1.7.3) package.

### MultiNicheNet analysis

MultiNicheNetR (V1.0.1)^63^ was used to identify differentially expressed active receptor–ligand interactions between broad and narrow neutralizers. The top 50 receptor–ligand interactions received by NK cells were identified using (logFC_threashold of 0.5, p_val_threashold of 0.05 and fraction_cutoff of 0.05). These receptor–ligand interactions were manually verified in the literature.

### Analysis of publicly available COVID-19 tissue datasets

AnnData or Seurat objects from Lindeboom et al. 2024 (ref. ^91^) and Walsh et al. 2025 (ref. ^90^) were downloaded from the COVID-19 Cell Atlas (http://www.covid19cellatlas.org) and Broad Institute Single Cell Atlas (http://www.singlecell.broadinstitute.org). Count matrix and metadata were extracted from the anndata object (Lindeboom et al. 2024 (ref. ^91^)), and CreateSeuratObject() was used with min.features = 500 and min.cells = 30. UpdateSeuratObject() was used to update Seurat object (Walsh et al. 2025 (ref. ^90^)) to V4.4.0. Seurat objects were subsetted to appropriate patients, time points and variants and processed as described above.

### Cell developmental potential score (cytoTRACE2)

CytoTRACE2 (V1.0.0) classifies the developmental potential of cells using an interpretable deep learning framework. Default parameters were used to score each NK cell using the cytotrace2() function on RNA counts using the ‘human’ model^55^.

### Primary cell isolation and culture

Healthy donor leukocyte reduction system chambers were obtained from the Stanford Blood Bank. All donors were younger than 50 years, with both male and female donors included. Leukocyte reduction system chambers were drained into 50-ml conicals and washed using at least 10 ml 1× PBS. Blood was divided equally among four 50-ml conicals, and 1× PBS was added up to 40 ml. 10 ml Ficoll (Cytiva; 17-1440-03) was layered underneath the blood, and tubes were spun at 2,000 rpm for 25 min with no brake. Buffy coat was removed using a manual transfer pipette. White blood cells were resuspended in 50 ml 1× PBS and spun down at 1,400 rpm for 10 min. If needed, the pellet was resuspended in 5 ml ACK lysis buffer (Invitrogen; A1049201) for 5 min. Cells were washed a total of three times in 50 ml PBS and resuspended at 20 × 10^6^ ml^−1^ in 10% DMSO and 90% FBS. Cells were frozen using ‘Mr. Frosty’ freezing containers overnight before long-term storage in liquid nitrogen. PBMCs were thawed at 37 °C and washed with complete RP10 (Roswell Park Memorial Institute medium (RPMI) (Gibco;11875093), 10% FBS, 1% Penicillin–Streptomycin–Amphotericin B, 1% L-glutamine, 10 mM HEPES, 1% NEAA, 1% sodium pyruvate) with 20 µl benzonase.

### NK isolation and activation

NK cells were isolated using Miltenyi NK Cell Isolation Kit, human (Miltenyi; 130-092-657), according to manufacturer’s protocol. After isolation, NK cells were incubated for 24 h with 5,000 U ml^−1^ rh-IFNα A/D (pbl; 11200-1) in complete RP10 in 96-well U-bottom plates. For analysis of NK migratory receptors, activated NK cells were washed in fluorescence-activated cell sorting (FACS) (1× PBS, 0.05% BSA, 2% FBS) twice and resuspended in ViaDye Red (Cytek; R7-60008) at 1:6,000 dilution in PBS. After 30 min at room temperature, cells were washed twice with FACS and resuspended in a cocktail of surface antibodies (anti-CX3CR1 BV421, anti-CD3 BV785, anti-CD14 BV785, anti-CD19 BV785, anti-CXCR5 Alexa Fluor 488, anti-CD56 PE-Cy7 and anti-CD16 700). After surface stain, cells were washed twice in FACS and fixed in 2% paraformaldehyde (EMS; 15710) for 10 min at room temperature and resuspended in FACS for analysis on a Cytek Aurora flow cytometer.

### iT_FH_-like cell differentiation

iT_FH_-like cells were generated on the basis of Schmitt et al. 2014 with some differences^95^. Naive CD4^+^ T cells were isolated from cryopreserved PBMCs with a Miltenyi Naive CD4^+^ T Cell Isolation Kit II, human (Miltenyi; 130-094-131), using 1.1× manufacturers recommendation for antibody cocktail, biotin beads and magnetic-activated cell sorting (MACS) buffer. Isolated cells were resuspended at 2 × 10^6^ cells per ml in complete RP10 and activated with 1 μg ml^−1^ SEB (Toxin Technologies; NC9442400) for 4 h at 37 °C in 96-well U-bottom plates. TGFβ (R&D Systems; 7754-BH-005, 5 ng ml^−1^) and IL-12 (R&D Systems; 219-IL-005, 1 ng ml^−1^) were added to the culture for 72 h. Cells were collected and stained for CXCR5 expression for 15 min at 37 °C by adding 20 μl anti-CXCR5 Alexa Fluor 488 to dry pellet. A total of 1 ml complete RP10 was added, and cells were spun down and resuspended in complete RPMI at 5–10 × 10^6^ cells per ml. CXCR5+ cells (iT_FH_-like cells) were sorted using Sony SH800 (Supplementary Fig. 4a) into complete RP10 with 50% FBS. Cells were counted to ensure viability. To analyze protein expression on unlabeled iT_FH_-like cells, after sorting, cells were washed in FACS twice and resuspended in ViaDye Red. After 30 min at room temperature, cells were washed twice with FACS and resuspended in a cocktail of surface antibodies (anti-CXCR5 Alexa Fluor 488, anti-PD-1 BV711, anti-ICOS BV711, anti-CD4 cfluor V610 and anti-CD40L PE-Cy7) for 30 min at room temperature. After surface stain, cells were washed twice in FACS and fixed in 2% paraformaldehyde for 10 min at room temperature. Cells were then washed twice in 1× permeabilization buffer (eBioscience; 00-8333-56) and stained with intracellular antibodies in 1× permeabilization buffer (anti-BCL6 PE) for 30 min at room temperature. Thereafter, cells were washed twice with permeabilization buffer, resuspended in FACS and analyzed on a Cytek Aurora flow cytometer.

### Isolation of activated and resting CD4^+^ T cells

CD4^+^ T cells were isolated from cryopreserved PBMCs using the Miltenyi CD4^+^ T Cell Isolation Kit, human (Miltenyi; 130-096-533), according to the manufacturer’s recommendation. Activated CD4^+^ T cells were resuspended at 2 × 10^6^ cells per ml in complete RP10 and with 1 μg ml^−1^ SEB for 72 h at 37 °C in 96-well U-bottom plates. Resting CD4^+^ T cells were isolated using the Miltenyi CD4^+^ T Cell Isolation Kit, human, immediately before killing assays were performed.

### Phenotyping NK ligands on iT_FH_-like, activated and resting CD4^+^ T cells

After iT_FH_-like cell differentiation and CD4^+^ T cell activation for 72 h, equal numbers of unsorted iT_FH_-like cells, activated or freshly isolated resting CD4^+^ T cells were plated. Cells were washed in FACS twice and resuspended in ViaDye Red. After 30 min at room temperature, cells were washed twice with FACS and resuspended in 2.5 μl-per-well Fc Block (Biolegend; 422302) diluted in FACS. After 10 min at room temperature, cells were spun down and resuspended in anti-CD4 cFluor V610, anti-CXCR5 Alexa Fluor 488 and one of anti-B7-H6 APC, anti-ULBP1 APC, anti-ULBP2-5-6 APC, anti-ULBP3 APC, anti-MICA APC, anti-MICB APC, anti-CD48 APC, anti-IgG1 APC, anti-IgG2a APC, anti-IgG2b APC or anti-IgGϰ APC for 30 min at room temperature. After surface stain, cells were washed twice in FACS and fixed in 2% paraformaldehyde for 10 min at room temperature. Fixed cells were resuspended in FACS and analyzed on a Cytek Aurora flow cytometer. For analysis, iT_FH_-like cells were gated as live, CD4^+^, CXCR5+ lymphocytes; CXCR5− cells were gated as live, CD4^+^, CXCR5− lymphocytes; activated CD4^+^ T cells were gated as live CD4^+^ cells; and resting CD4^+^ T cells were gated as live CD4^+^ cells (Extended Data Fig. 6e and Supplementary Fig. 4). Gates for ligand-positive cells were set using appropriate isotype controls for each ligand antibody and CD4^+^ T cell type.

### NK cell functional coculture and phenotyping with iT_FH_-like or activated CD4^+^ T cells

Target cells (iT_FH_-like or activated CD4^+^ T cells) were labeled with CellTrace Violet (Invitrogen; C34557) for 20 min at 37 °C at 1:10,000 dilution in PBS at a cell concentration of 1 × 10^6^ cells per ml. After labeling, 5× volume complete RPMI was added for 5 min to remove any free dye. Cells were then pelleted, washed once with complete RP10 and resuspended for coculture. NK cells and target cells were added at an E:T ratio of 1:5 in 96-well U-bottom plates. Cocultures were centrifuged at 1,000 rpm for 1 min to bring cells together and incubated overnight before analysis of NK cells by flow cytometry. A total of 4 h before the end of coculture, brefeldin A (eBioscience; 00-4506-51), monensin (eBioscience; 00-4501-51) and anti-CD107a phycoerythrin was added to the culture, followed by centrifugation at 1,000 rpm for 1 min. When coculture was complete, cells were washed in FACS twice and resuspended in ViaDye Red. After 30 min at room temperature, cells were washed twice with FACS and resuspended in a cocktail of surface antibodies (anti-NKG2D BV650, anti-CD38 Super Bright 600, anti-CD3 BV785, anti-CD14 BV785, anti-CD19 BV785, anti-CD69 PE-Dazzle594, anti-CD56 PE-Cy7, anti-TRAIL APC and anti-CD16 Alexa Fluor 700) in 1× Brilliant Stain Buffer (BD; 563794) diluted with FACS for another 30 min at room temperature. After surface stain, cells were washed twice in FACS and fixed in 2% paraformaldehyde for 10 min at room temperature. Cells were then washed twice in 1× permeabilization buffer and stained with intracellular antibodies (anti-perforin BV510, anti-IFNγ BV711 and anti-granzyme B PerCP-Cy5.5) in 1× Brilliant Stain Buffer and 1× permeabilization buffer diluted in water for 30 min at room temperature. Cells were then washed twice with permeabilization buffer, resuspended in FACS and analyzed on a Cytek Aurora flow cytometer.

### Flow cytometry-based NK cell killing assay against iT_FH_-like, activated CD4^+^ T and resting CD4^+^ T cells

Target cells (iT_FH_-like, activated or resting CD4^+^ T cells) were labeled with CellTrace Violet as described above. NK cells and target cells were added at an E:T ratio of 5:1 in 96-well U-bottom plates. Cells were centrifuged at 1,000 rpm for 1 min to bring cells together and incubated at 37 °C for 3 h. Cells were then washed twice in FACS and resuspended in ViaDye Red. Cells were then washed twice in FACS and fixed in 2% paraformaldehyde for 10 min at room temperature and resuspended in FACS for analysis on a Cytek Aurora flow cytometer.

### Blocking NKG2D and NKp30 in flow cytometry-based killing assays against iT_FH_-like cells

iT_FH_-like cells were labeled with CellTrace Violet as described above. NK cells were pre-incubated with anti-NKG2D^116^ (10 µg ml^−1^) and/or anti-NKp30^117^ (10 µg ml^−1^)-blocking antibodies for 1 h at 37 °C. Isotype control wells contained nonspecific binding IgG1 and IgG2a (10 µg ml^−1^). iT_FH_-like target cells were added at an E:T ratio of 5:1 in 96-well U-bottom plates. Cells were centrifuged at 1,000 rpm for 1 min to bring cells together and incubated at 37 °C for 3 h. Cells were then washed twice in FACS and resuspended in ViaDye Red. Cells were then washed twice in FACS and fixed in 2% paraformaldehyde for 10 min at room temperature and resuspended in FACS for analysis on a Cytek Aurora flow cytometer.

### Coculture of NK, iT_FH_-like and B cells

NK:iT_FH_:B cell coculture was developed per Bradley et al. 2018 (ref. ^28^), with some modifications. iT_FH_-like cells were isolated and differentiated as described above. NK cells were activated with IFNα as described above. A total of 30 × 10^3^ iT_FH_-like cells and 15 × 10^4^ NK cells per well were plated in 96-well U-bottom plates and spun down at 1,000 rpm for 1 min and placed at 37 °C for 1 h while B cells were isolated. B cells were isolated from cryopreserved PBMCs using a Miltenyi B cell Isolation Kit II, human (Miltenyi; 130-091-151), according to the manufacturer’s recommendation. In total, 60 × 10^3^ B cells were added to the coculture, and the plate was spun down at 1,000 rpm for 1 min. After 6 days of NK:iT_FH_-like:B cell coculture, supernatants were collected for IgM and IgG ELISAs. Cells were washed in FACS twice and resuspended in ViaDye Red. After 30 min at room temperature, cells were washed twice with FACS and resuspended in a cocktail of surface antibodies (anti-CD4 cfluor V610, anti-CD38 SuperBright600, anti-CD3 BV785, anti-IgM PE, anti-CD19 PerCP-Cy5.5, anti-CD56 PE-Cy7, anti-CD27 APC and anti-CD16 AlexaFluor700) in 1× Brilliant Stain Buffer (BD; 563794) diluted with FACS for another 30 min at room temperature. After surface stain, cells were washed twice in FACS and fixed in 2% paraformaldehyde for 10 min at room temperature. Cells were then resuspended in 250 µl FACS and 50 µl Precision Count Beads (Biolegend; 424902) for analysis on Cytek Aurora. Supernatant levels of IgM and IgG were evaluated using an IgM Human ELISA Kit (Thermo Scientific; BMS2098) and IgG (total) Human ELISA Kit (Thermo Scientific; BMS2091) according to the manufacturer’s recommendations.

### qPCR

After NK cells were isolated and activated with IFNα as described above for 24 h, cells were lysed in DNA/RNA Shield (Zymo Research; R1100-250). RNA was isolated using RNA Clean and Concentrator and Concentrator kits (Zymo Research; R1018), and excess DNA was removed from the samples using the TURBO DNA-free Kit according to the manufacturer’s instructions (Fisher Scientific; cat. no. AM1907). Reverse transcription qPCR reactions were prepared with 1 ng RNA per well using the Invitrogen superscript III Platinum One Step qRT PCR Kit with ROX (Invitrogen; 11745500) and TaqMan Gene Expression assay FAM (Thermo Scientific; ISG15; 4331182, MX1; 4331182, OAS1; 4331182, IFIT1; 4331182) according to the manufacturer’s protocol. QuantStudio 3 Real-Time PCR System was used to quantify transcript levels (ThermoFisher; A28567). Three technical replicates of each sample were measured, and all samples were normalized to an 18S endogenous control (Thermo Scientific; 4352930E). ΔCt was calculated as Ct (experimental gene) − Ct (18S). ΔΔCt was calculated as ΔCt(unstimulated NK cells) − ΔCt(IFNα-activated NK cells).

### Flow cytometry analysis

Spectral unmixing was performed in Cytek Spectroflo Software using UltraComp eBeads Plus Compensation Beads (Thermo Scientific; 01-3333-42) as single color controls. Fluorescence-minus-one validation was completed for each flow panel. Unmixed flow cytometry standard (FCS) files were imported to FlowJo V10.10.0 and gated as described in Supplementary Figs. 3–5 to identify cell populations and marker expression.

### Data visualization

All data analyses and visualizations were performed in the open-source software R (V4.2.2)^118^. The R package Seurat (V4.4.0) was used to generate UMAP projections. ComplexHeatmap (V2.14.0) was used for all heatmaps^119,120^. ComplexUpset (V4.2.2) was used to create UpSet plots^121^. MultiNicheNetR was used to create circos plots. EnhancedVolcanoPlot (V1.16.0) was used to draw volcano plots. Colors for plots were generated using MoMAColors, MetBrewer, NatParkPalettes and PNWColors. Custom ggplot2 functions were used for all other plots.

### Reporting summary

Further information on research design is available in the Nature Portfolio Reporting Summary linked to this article.

## Online content

Any methods, additional references, Nature Portfolio reporting summaries, source data, extended data, supplementary information, acknowledgements, peer review information; details of author contributions and competing interests; and statements of data and code availability are available at 10.1038/s41590-025-02341-1.

## Supplementary information

Supplementary Information Supplementary Figs. 1–5 and consortium members.

Reporting Summary

Supplementary Table 1 De-identified clinical and demographic metadata for each participant in scRNA-seq and CyTOF.

Supplementary Table 2 Significant differentially expressed genes for each cell type between broad and narrow neutralizers by Seurat’s FindMarkers(). Significantly enriched KEGG pathways for each cell type for genes upregulated in broad or narrow neutralizers. Genes significantly correlated with breadth score for whole PBMCs and NK cells using pseudobulk correlation analysis with DESeq2. Sheet names indicate cell types, methods and breadth groups.

Supplementary Table 3 Flow cytometry and CyTOF reagents.

Supplementary Table 4 Raw neutralization assay data.

Supplementary Table 5 Raw qPCR data.

## Source data

Source Data Fig. 1 NT50 data.

Source Data Fig. 3 Narrow neutralizer KEGG pathways enriched in each cell type.

Source Data Fig. 6 Cell–cell communication results.

Source Data Fig. 7 qPCR and flow cytometry data.

Source Data Fig. 8 Flow cytometry and ELISA data.

Source Data Extended Data Fig. 4 Flow cytometry data.

Source Data Extended Data Fig. 5 Flow cytometry data.

Source Data Extended Data Fig. 6 Flow cytometry data.

## Data Availability

scRNA-seq and CyTOF data were originally published by Wilk et al. in 2021 (ref. ^30^). FCS files (mass cytometry) with de-identified metadata supporting this publication are available at ImmPort (https://www.immport.org) under study accession no. SDY1708. Data from scRNA-seq have been deposited with the Gene Expression Omnibus under accession no. GSE174072. FCS flow cytometry files from in vitro assays are deposited at CytoBank Community Experiment (ID no. 123058). Source data are provided with this paper.

## Code availability

All code will be available upon publication via GitHub at https://github.com/BlishLab/SARSCoV2_Antibody_Breadth.

## References

[1] Yuki, K., Fujiogi, M. & Koutsogiannaki, S. COVID-19 pathophysiology: a review. *Clin. Immunol.* **215**, 108427 (2020). 32325252 10.1016/j.clim.2020.108427 PMC7169933

[2] *WHO COVID-19 Dashboard: COVID-19 Deaths* (WHO, accessed 20 October 2025); https://data.who.int/dashboards/covid19/deaths?n=o

[3] COVID-19 Forecasting Team. Past SARS-CoV-2 infection protection against re-infection: a systematic review and meta-analysis. *Lancet* **401**, 833–842 (2023). 10.1016/S0140-6736(22)02465-5 PMC9998097 36930674

[4] Tao, K. et al. The biological and clinical significance of emerging SARS-CoV-2 variants. *Nat. Rev. Genet.* **22**, 757–773 (2021). 34535792 10.1038/s41576-021-00408-x PMC8447121

[5] Planas, D. et al. Considerable escape of SARS-CoV-2 omicron to antibody neutralization. *Nature* **602**, 671–675 (2022). 35016199 10.1038/s41586-021-04389-z

[6] Planas, D. et al. Reduced sensitivity of SARS-CoV-2 variant delta to antibody neutralization. *Nature* **596**, 276–280 (2021). 34237773 10.1038/s41586-021-03777-9

[7] Rydyznski, C. E. & Waggoner, S. N. Boosting vaccine efficacy the natural (killer) way. *Trends Immunol.* **36**, 536–546 (2015). 26272882 10.1016/j.it.2015.07.004 PMC4567442

[8] Cox, A. et al. Targeting natural killer cells to enhance vaccine responses. *Trends Pharmacol. Sci.* **42**, 789–801 (2021). 34311992 10.1016/j.tips.2021.06.004 PMC8364504

[9] Coccia, M. et al. Cellular and molecular synergy in AS01-adjuvanted vaccines results in an early IFNγ response promoting vaccine immunogenicity. *npj Vaccines* **2**, 25 (2017). 29263880 10.1038/s41541-017-0027-3 PMC5627273

[10] Kazmin, D. et al. Systems analysis of protective immune responses to RTS,S malaria vaccination in humans. *Proc. Natl Acad. Sci. USA* **114**, 2425–2430 (2017). 28193898 10.1073/pnas.1621489114 PMC5338562

[11] Muyanja, E. et al. Immune activation alters cellular and humoral responses to yellow fever 17D vaccine. *J. Clin. Invest.* **124**, 3147–3158 (2014). 24911151 10.1172/JCI75429 PMC4071376

[12] Rydyznski, C. E. et al. Affinity maturation is impaired by natural killer cell suppression of germinal centers. *Cell Rep.* **24**, 3367–3373 (2018). 30257198 10.1016/j.celrep.2018.08.075 PMC6192537

[13] Rydyznski, C. et al. Generation of cellular immune memory and B-cell immunity is impaired by natural killer cells. *Nat. Commun.* **6**, 6375 (2015). 25721802 10.1038/ncomms7375 PMC4346304

[14] Gyurova, I. E., Ali, A. & Waggoner, S. N. Natural killer cell regulation of B cell responses in the context of viral infection. *Viral Immunol.* **33**, 334–341 (2020). 31800366 10.1089/vim.2019.0129 PMC7247022

[15] Waggoner, S. N., Cornberg, M., Selin, L. K. & Welsh, R. M. Natural killer cells act as rheostats modulating antiviral T cells. *Nature* **481**, 394–398 (2012). 10.1038/nature10624 PMC3539796 22101430

[16] Cook, K. D., Waggoner, S. N. & Whitmire, J. K. NK cells and their ability to modulate T cells during virus infections. *Crit. Rev. Immunol.* **34**, 359–388 (2014). 25404045 10.1615/critrevimmunol.2014010604 PMC4266186

[17] Waggoner, S. N. et al. Roles of natural killer cells in antiviral immunity. *Curr. Opin. Virol.* **16**, 15–23 (2016). 26590692 10.1016/j.coviro.2015.10.008 PMC4821726

[18] Mot, L. D. et al. Transcriptional profiles of adjuvanted hepatitis B vaccines display variable interindividual homogeneity but a shared core signature. *Sci. Transl. Med.* **12**, eaay8618 (2020). 33177181 10.1126/scitranslmed.aay8618

[19] Peppa, D. et al. Up-regulation of a death receptor renders antiviral T cells susceptible to NK cell–mediated deletion. *J. Exp. Med.* **210**, 99–114 (2013). 23254287 10.1084/jem.20121172 PMC3549717

[20] Lang, P. A. et al. Natural killer cell activation enhances immune pathology and promotes chronic infection by limiting CD8^+^ T-cell immunity. *Proc. Natl Acad. Sci. USA* **109**, 1210–1215 (2012). 22167808 10.1073/pnas.1118834109 PMC3268324

[21] Xu, H. C. et al. Type I interferon protects antiviral CD8^+^ T cells from NK cell cytotoxicity. *Immunity* **40**, 949–960 (2014). 24909887 10.1016/j.immuni.2014.05.004

[22] Schuster, I. S. et al. TRAIL + NK cells control CD4+ T cell responses during chronic viral infection to limit autoimmunity. *Immunity* **41**, 646–656 (2014). 25367576 10.1016/j.immuni.2014.09.013

[23] Rabinovich, B. A. et al. Activated, but not resting, T cells can be recognized and killed by syngeneic NK cells. *J. Immunol.* **170**, 3572–3576 (2003). 12646619 10.4049/jimmunol.170.7.3572

[24] Ferlazzo, G. & Morandi, B. Cross-talks between natural killer cells and distinct subsets of dendritic cells. *Front. Immunol.* **5**, 159 (2014). 24782864 10.3389/fimmu.2014.00159 PMC3989561

[25] Ferlazzo, G. & Moretta, L. Dendritic cell editing by natural killer cells. *Crit. Rev. Oncog.* **19**, 67–75 (2014). 24941374 10.1615/critrevoncog.2014010827

[26] Storkus, W. J. & Dawson, J. R. B cell sensitivity to natural killing: correlation with target cell stage of differentiation and state of activation. *J. Immunol.* **136**, 1542–1547 (1986). 3081629

[27] Brieva, J. A., Targan, S. & Stevens, R. H. NK and T cell subsets regulate antibody production by human in vivo antigen-induced lymphoblastoid B cells. *J. Immunol.* **132**, 611–615 (1984). 6228592

[28] Bradley, T. et al. RAB11FIP5 expression and altered natural killer cell function are associated with induction of HIV broadly neutralizing antibody responses. *Cell* **175**, 387–399 (2018). 30270043 10.1016/j.cell.2018.08.064 PMC6176872

[29] Wilk, A. J. et al. A single-cell atlas of the peripheral immune response in patients with severe COVID-19. *Nat. Med.* **26**, 1070–1076 (2020). 32514174 10.1038/s41591-020-0944-y PMC7382903

[30] Wilk, A. J. et al. Multi-omic profiling reveals widespread dysregulation of innate immunity and hematopoiesis in COVID-19. *J. Exp. Med.* **218**, e20210582 (2021). 34128959 10.1084/jem.20210582 PMC8210586

[31] Tan, C. W. et al. A SARS-CoV-2 surrogate virus neutralization test based on antibody-mediated blockage of ACE2–spike protein–protein interaction. *Nat. Biotechnol.* **38**, 1073–1078 (2020). 32704169 10.1038/s41587-020-0631-z

[32] Love, M. I., Huber, W. & Anders, S. Moderated estimation of fold change and dispersion for RNA-seq data with DESeq2. *Genome Biol.* **15**, 550 (2014). 25516281 10.1186/s13059-014-0550-8 PMC4302049

[33] Rusinova, I. et al. INTERFEROME v2.0: an updated database of annotated interferon-regulated genes. *Nucleic Acids Res.* **41**, D1040–D1046 (2012). 23203888 10.1093/nar/gks1215 PMC3531205

[34] Hamann, I. et al. Analyses of phenotypic and functional characteristics of CX3CR1-expressing natural killer cells. *Immunology* **133**, 62–73 (2011). 21320123 10.1111/j.1365-2567.2011.03409.x PMC3088968

[35] Szklarczyk, D. et al. STRING v10: protein–protein interaction networks, integrated over the tree of life. *Nucleic Acids Res.* **43**, D447–D452 (2015). 25352553 10.1093/nar/gku1003 PMC4383874

[36] Beisser, D., Klau, G. W., Dandekar, T., Müller, T. & Dittrich, M. T. BioNet: an R-Package for the functional analysis of biological networks. *Bioinformatics* **26**, 1129–1130 (2010). 20189939 10.1093/bioinformatics/btq089

[37] Dittrich, M. T., Klau, G. W., Rosenwald, A., Dandekar, T. & Müller, T. Identifying functional modules in protein–protein interaction networks: an integrated exact approach. *Bioinformatics* **24**, i223–i231 (2008). 18586718 10.1093/bioinformatics/btn161 PMC2718639

[38] Baranovskyi, T., Osypchuk, D., Bezpala, A. & Dons’koi, B. CD8αα expression on NK cells is associated with different K562 and MOLT4 killing capabilities of PBMC. *J. Allergy Clin. Immunol.* **149**, AB197 (2022).

[39] Germain, C. et al. Induction of lectin-like transcript 1 (LLT1) protein cell surface expression by pathogens and interferon-γ contributes to modulate immune responses. *J. Biol. Chem.* **286**, 37964–37975 (2011). 21930700 10.1074/jbc.M111.285312 PMC3207416

[40] Borrego, F., Masilamani, M., Kabat, J., Sanni, T. B. & Coligan, J. E. The cell biology of the human natural killer cell CD94/NKG2A inhibitory receptor. *Mol. Immunol.* **42**, 485–488 (2005). 15607803 10.1016/j.molimm.2004.07.031

[41] Vivier, E., Tomasello, E., Baratin, M., Walzer, T. & Ugolini, S. Functions of natural killer cells. *Nat. Immunol.* **9**, 503–510 (2008). 18425107 10.1038/ni1582

[42] Hao, Y. et al. Integrated analysis of multimodal single-cell data. *Cell* **184**, 3573–3587 (2021). 34062119 10.1016/j.cell.2021.04.048 PMC8238499

[43] Braud, V. M. et al. HLA-E binds to natural killer cell receptors CD94/NKG2A, B and C. *Nature* **391**, 795–799 (1998). 9486650 10.1038/35869

[44] Flores-Villanueva, P. O. et al. Control of HIV-1 viremia and protection from AIDS are associated with HLA-Bw4 homozygosity. *Proc. Natl Acad. Sci. USA* **98**, 5140–5145 (2001). 11309482 10.1073/pnas.071548198 PMC33177

[45] Maruthamuthu, S. et al. Individualized constellation of killer cell immunoglobulin-like receptors and cognate HLA class I ligands that controls natural killer cell antiviral immunity predisposes COVID-19. *Front. Genet.* **13**, 845474 (2022). 35273641 10.3389/fgene.2022.845474 PMC8902362

[46] Kanehisa, M. & Goto, S. KEGG: Kyoto Encyclopedia of Genes and Genomes. *Nucleic Acids Res.* **28**, 27–30 (2000). 10592173 10.1093/nar/28.1.27 PMC102409

[47] Schlums, H. et al. Cytomegalovirus infection drives adaptive epigenetic diversification of NK cells with altered signaling and effector function. *Immunity* **42**, 443–456 (2015). 25786176 10.1016/j.immuni.2015.02.008 PMC4612277

[48] Strauss-Albee, D. M. et al. Human NK cell repertoire diversity reflects immune experience and correlates with viral susceptibility. *Sci. Transl. Med.* **7**, 297ra115 (2015). 26203083 10.1126/scitranslmed.aac5722 PMC4547537

[49] Lutz, C. S., Schleiss, M. R., Fowler, K. B. & Lanzieri, T. M. Updated national and state-specific prevalence of congenital cytomegalovirus infection, United States, 2018–2022. *J. Public Health Manag. Pract.* **31**, 234–243 (2025). 39231390 10.1097/PHH.0000000000002043 PMC12068531

[50] Osman, M. et al. Impaired natural killer cell counts and cytolytic activity in patients with severe COVID-19. *Blood Adv.* **4**, 5035–5039 (2020). 33075136 10.1182/bloodadvances.2020002650 PMC7594380

[51] Krämer, B. et al. Early IFN-α signatures and persistent dysfunction are distinguishing features of NK cells in severe COVID-19. *Immunity* **54**, 2650–2669 (2021). 34592166 10.1016/j.immuni.2021.09.002 PMC8416549

[52] Witkowski, M. et al. Untimely TGFβ responses in COVID-19 limit antiviral functions of NK cells. *Nature* **600**, 295–301 (2021). 34695836 10.1038/s41586-021-04142-6

[53] Lee, M. J. & Blish, C. A. Defining the role of natural killer cells in COVID-19. *Nat. Immunol.* **24**, 1628–1638 (2023). 37460639 10.1038/s41590-023-01560-8 PMC10538371

[54] Leem, G. et al. Abnormality in the NK cell population is prolonged in severe COVID-19 patients. *J. Allergy Clin. Immun.* **148**, 996–1006.e18 (2021). 34339730 10.1016/j.jaci.2021.07.022 PMC8324384

[55] Kang, M. et al. Improved reconstruction of single-cell developmental potential with CytoTRACE 2. *Nat. Methods*10.1038/s41592-025-02857-2 (2025). 10.1038/s41592-025-02857-2 PMC12615260 41145665

[56] Lee, M. J. et al. NK cell–monocyte cross-talk underlies NK cell activation in severe COVID-19. *J. Immunol.* **212**, 1693–1705 (2024). 38578283 10.4049/jimmunol.2300731 PMC11102029

[57] Aldemir, H. et al. Cutting edge: lectin-like transcript 1 is a ligand for the CD161 receptor. *J. Immunol.* **175**, 7791–7795 (2005). 16339512 10.4049/jimmunol.175.12.7791

[58] Rosen, D. B. et al. Cutting edge: lectin-like transcript-1 is a ligand for the inhibitory human NKR-P1A receptor. *J. Immunol.* **175**, 7796–7799 (2005). 16339513 10.4049/jimmunol.175.12.7796

[59] Raulet, D. H. Roles of the NKG2D immunoreceptor and its ligands. *Nat. Rev. Immunol.* **3**, 781–790 (2003). 14523385 10.1038/nri1199

[60] Bottino, C., Castriconi, R., Moretta, L. & Moretta, A. Cellular ligands of activating NK receptors. *Trends Immunol.* **26**, 221–226 (2005). 15797513 10.1016/j.it.2005.02.007

[61] Bottino, C. et al. Identification of PVR (CD155) and nectin-2 (CD112) as cell surface ligands for the human DNAM-1 (CD226) activating molecule. *J. Exp. Med.* **198**, 557–567 (2003). 12913096 10.1084/jem.20030788 PMC2194180

[62] Fuchs, A., Cella, M., Giurisato, E., Shaw, A. S. & Colonna, M. Cutting edge: CD96 (tactile) promotes NK cell–target cell adhesion by interacting with the poliovirus receptor (CD155). *J. Immunol.* **172**, 3994–3998 (2004). 15034010 10.4049/jimmunol.172.7.3994

[63] Browaeys, R. et al. MultiNicheNet: a flexible framework for differential cell–cell communication analysis from multi-sample multi-condition single-cell transcriptomics data. Preprint at *bioRxiv*10.1101/2023.06.13.544751 (2023).

[64] Peng, P. et al. TGFBI secreted by tumor-associated macrophages promotes glioblastoma stem cell-driven tumor growth via integrin αvβ5-Src-Stat3 signaling. *Theranostics* **12**, 4221–4236 (2022). 35673564 10.7150/thno.69605 PMC9169371

[65] Qing, J. et al. Transforming growth factor β/Smad3 signaling regulates IRF-7 function and transcriptional activation of the beta interferon promoter. *Mol. Cell. Biol.* **24**, 1411–1425 (2004). 14729983 10.1128/MCB.24.3.1411-1425.2004 PMC321430

[66] Pham, A. H. et al. Human cytomegalovirus blocks canonical TGFβ signaling during lytic infection to limit induction of type I interferons. *PLoS Pathog.* **17**, e1009380 (2021). 34411201 10.1371/journal.ppat.1009380 PMC8407580

[67] Pahima, H., Puzzovio, P. G. & Levi-Schaffer, F. 2B4 and CD48: a powerful couple of the immune system. *Clin. Immunol.* **204**, 64–68 (2019). 30366105 10.1016/j.clim.2018.10.014

[68] Wang, T. et al. SECTM1 produced by tumor cells attracts human monocytes via CD7-mediated activation of the PI3K pathway. *J. Investig. Dermatol.* **134**, 1108–1118 (2014). 24157461 10.1038/jid.2013.437 PMC3961532

[69] Kumaresan, P. R., Lai, W. C., Chuang, S. S., Bennett, M. & Mathew, P. A. CS1, a novel member of the CD2 family, is homophilic and regulates NK cell function. *Mol. Immunol.* **39**, 1–8 (2002). 12213321 10.1016/s0161-5890(02)00094-9

[70] Wu, N. & Veillette, A. SLAM family receptors in normal immunity and immune pathologies. *Curr. Opin. Immunol.* **38**, 45–51 (2016). 26682762 10.1016/j.coi.2015.11.003

[71] Takasaki, S., Hayashida, K., Morita, C., Ishibashi, H. & Niho, Y. cd56 directly interacts in the process of NCAM-positive target cell-killing by NK cells. *Cell Biol. Int.* **24**, 101–108 (2000). 10772769 10.1006/cbir.1999.0457

[72] Lee, N., Ishitani, A. & Geraghty, D. E. HLA-F is a surface marker on activated lymphocytes. *Eur. J. Immunol.* **40**, 2308–2318 (2010). 20865824 10.1002/eji.201040348 PMC3867582

[73] Zhang, Y.-L. et al. SPON2 promotes M1-like macrophage recruitment and inhibits hepatocellular carcinoma metastasis by distinct integrin–rho GTPase–hippo pathways. *Cancer Res.* **78**, 2305–2317 (2018). 29440144 10.1158/0008-5472.CAN-17-2867

[74] Toyofuku, T. et al. Semaphorin-4A, an activator for T-cell-mediated immunity, suppresses angiogenesis via Plexin-D1. *EMBO J.* **26**, 1373–1384 (2007). 17318185 10.1038/sj.emboj.7601589 PMC1817636

[75] Cheng, G. et al. Identification of PLXDC1 and PLXDC2 as the transmembrane receptors for the multifunctional factor PEDF. *eLife* **3**, e05401 (2014). 25535841 10.7554/eLife.05401 PMC4303762

[76] Hò, G.-G. T. et al. NKG2A/CD94 is a new immune receptor for HLA-G and distinguishes amino acid differences in the HLA-G heavy chain. *Int. J. Mol. Sci.* **21**, 4362 (2020). 32575403 10.3390/ijms21124362 PMC7352787

[77] Kang, X. et al. Inhibitory leukocyte immunoglobulin-like receptors: immune checkpoint proteins and tumor sustaining factors. *Cell Cycle* **15**, 25–40 (2016). 26636629 10.1080/15384101.2015.1121324 PMC4825776

[78] Lin, A. & Yan, W.-H. The emerging roles of human leukocyte antigen-F in immune modulation and viral infection. *Front. Immunol.* **10**, 964 (2019). 31134067 10.3389/fimmu.2019.00964 PMC6524545

[79] Jones, D. C. et al. HLA class I allelic sequence and conformation regulate leukocyte Ig-like receptor binding. *J. Immunol.* **186**, 2990–2997 (2011). 21270408 10.4049/jimmunol.1003078

[80] Lepin, E. J. M. et al. Functional characterization of HLA-F and binding of HLA-F tetramers to ILT2 and ILT4 receptors. *Eur. J. Immunol.* **30**, 3552–3561 (2000). 11169396 10.1002/1521-4141(200012)30:12<3552::AID-IMMU3552>3.0.CO;2-L

[81] Moiseeva, E. P., Williams, B., Goodall, A. H. & Samani, N. J. Galectin-1 interacts with β-1 subunit of integrin. *Biochem. Biophys. Res. Commun.* **310**, 1010–1016 (2003). 14550305 10.1016/j.bbrc.2003.09.112

[82] Gupta, G. S. in *Animal Lectins: Form, Function and Clinical Applications* 991–1026 (Springer, 2012).

[83] Carlow, D. A. et al. PSGL-1 function in immunity and steady state homeostasis. *Immunol. Rev.* **230**, 75–96 (2009). 19594630 10.1111/j.1600-065X.2009.00797.x

[84] Zheng, P.-S. et al. PG-M/versican binds to P-selectin glycoprotein ligand-1 and mediates leukocyte aggregation. *J. Cell Sci.* **117**, 5887–5895 (2004). 15522894 10.1242/jcs.01516

[85] Seo, D.-W. et al. Shp-1 mediates the antiproliferative activity of tissue inhibitor of metalloproteinase-2 in human microvascular endothelial cells. *J. Biol. Chem.* **281**, 3711–3721 (2006). 16326706 10.1074/jbc.M509932200 PMC1361361

[86] Wöhner, B. et al. Proteolysis of CD44 at the cell surface controls a downstream protease network. *Front. Mol. Biosci.* **10**, 1026810 (2023). 36876041 10.3389/fmolb.2023.1026810 PMC9981664

[87] Li, B. et al. Cis interactions between CD2 and its ligands on T cells are required for T cell activation. *Sci. Immunol.* **7**, eabn6373 (2022). 35930657 10.1126/sciimmunol.abn6373

[88] Lu, J. et al. Expansion of circulating T follicular helper cells is associated with disease progression in HIV-infected individuals. *J. Infect. Public Health* **11**, 685–690 (2018). 29409739 10.1016/j.jiph.2018.01.005

[89] Thevarajan, I. et al. Breadth of concomitant immune responses prior to patient recovery: a case report of non-severe COVID-19. *Nat. Med.* **26**, 453–455 (2020). 32284614 10.1038/s41591-020-0819-2 PMC7095036

[90] Walsh, J. M. L. et al. Variants and vaccines impact nasal immunity over three waves of SARS-CoV-2. *Nat. Immunol.* **26**, 294–307 (2025). 39833605 10.1038/s41590-024-02052-z PMC13052507

[91] Lindeboom, R. G. H. et al. Human SARS-CoV-2 challenge uncovers local and systemic response dynamics. *Nature* **631**, 189–198 (2024). 38898278 10.1038/s41586-024-07575-x PMC11222146

[92] Cousens, L. P. et al. Two roads diverged: interferon α/β– and interleukin 12–mediated pathways in promoting T Cell interferon γ responses during viral infection. *J. Exp. Med.* **189**, 1315–1328 (1999). 10209048 10.1084/jem.189.8.1315 PMC2193028

[93] Nguyen, K. B. et al. Critical role for STAT4 activation by type 1 interferons in the interferon-γ response to viral infection. *Science* **297**, 2063–2066 (2002). 12242445 10.1126/science.1074900

[94] Levy, D. E., Marié, I. J. & Durbin, J. E. Induction and function of type I and III interferon in response to viral infection. *Curr. Opin. Virol.* **1**, 476–486 (2011). 22323926 10.1016/j.coviro.2011.11.001 PMC3272644

[95] Schmitt, N. et al. The cytokine TGF-β co-opts signaling via STAT3-STAT4 to promote the differentiation of human T_FH_ cells. *Nat. Immunol.* **15**, 856–865 (2014). 25064073 10.1038/ni.2947 PMC4183221

[96] Crotty, S. T. Follicular helper cell differentiation, function, and roles in disease. *Immunity* **41**, 529–542 (2014). 25367570 10.1016/j.immuni.2014.10.004 PMC4223692

[97] Vivier, E. et al. Innate or adaptive immunity? The example of natural killer cells. *Science* **331**, 44–49 (2011). 21212348 10.1126/science.1198687 PMC3089969

[98] Nakajima, H., Cella, M., Langen, H., Friedlein, A. & Colonna, M. Activating interactions in human NK cell recognition: the role of 2B4-CD48. *Eur. J. Immunol.* **29**, 1676–1683 (1999). 10359122 10.1002/(SICI)1521-4141(199905)29:05<1676::AID-IMMU1676>3.0.CO;2-Y

[99] Brandt, C. S. et al. The B7 family member B7-H6 is a tumor cell ligand for the activating natural killer cell receptor NKp30 in humans. *J. Exp. Med.* **206**, 1495–1503 (2009). 19528259 10.1084/jem.20090681 PMC2715080

[100] Quatrini, L. et al. Ubiquitin-dependent endocytosis of NKG2D-DAP10 receptor complexes activates signaling and functions in human NK cells. *Sci. Signal.* **8**, ra108 (2015). 26508790 10.1126/scisignal.aab2724

[101] Schoggins, J. W. Interferon-stimulated genes: what do they all do?.*Annu. Rev. Virol.* **6**, 567–584 (2019). 31283436 10.1146/annurev-virology-092818-015756

[102] Hadjadj, J. et al. Impaired type I interferon activity and inflammatory responses in severe COVID-19 patients. *Science* **369**, 718–724 (2020). 32661059 10.1126/science.abc6027 PMC7402632

[103] Singh, D. K. et al. Myeloid cell interferon responses correlate with clearance of SARS-CoV-2. *Nat. Commun.* **13**, 679 (2022). 35115549 10.1038/s41467-022-28315-7 PMC8814034

[104] Blanco-Melo, D. et al. Imbalanced host response to SARS-CoV-2 drives development of COVID-19. *Cell* **181**, 1036–1045 (2020). 32416070 10.1016/j.cell.2020.04.026 PMC7227586

[105] Victora, G. D. et al. Germinal center dynamics revealed by multiphoton microscopy with a photoactivatable fluorescent reporter. *Cell* **143**, 592–605 (2010). 21074050 10.1016/j.cell.2010.10.032 PMC3035939

[106] Guo, A.-L. et al. Implications of the accumulation of CXCR5^+^ NK cells in lymph nodes of HIV-1 infected patients. *EBioMedicine* **75**, 103794 (2021). 34973625 10.1016/j.ebiom.2021.103794 PMC8728057

[107] Huot, N. et al. Natural killer cells migrate into and control simian immunodeficiency virus replication in lymph node follicles in African green monkeys. *Nat. Med.* **23**, 1277–1286 (2017). 29035370 10.1038/nm.4421 PMC6362838

[108] Hammer, Q. et al. SARS-CoV-2 Nsp13 encodes for an HLA-E-stabilizing peptide that abrogates inhibition of NKG2A-expressing NK cells. *Cell Rep.* **38**, 110503 (2022). 35235832 10.1016/j.celrep.2022.110503 PMC8858686

[109] Graydon, E. K. et al. Natural killer cells and BNT162b2 mRNA vaccine reactogenicity and durability. *Front. Immunol.* **14**, 1225025 (2023). 37711632 10.3389/fimmu.2023.1225025 PMC10497936

[110] Polack, F. P. et al. Safety and efficacy of the BNT162b2 mRNA COVID-19 vaccine. *N. Engl. J. Med.* **383**, 2603–2615 (2020). 33301246 10.1056/NEJMoa2034577 PMC7745181

[111] Andrews, N. et al. COVID-19 vaccine effectiveness against the omicron (B.1.1.529) variant. *N. Engl. J. Med.* **386**, 1532–1546 (2022). 35249272 10.1056/NEJMoa2119451 PMC8908811

[112] VanBlargan, L. A. et al. An infectious SARS-CoV-2 B.1.1.529 omicron virus escapes neutralization by therapeutic monoclonal antibodies. *Nat. Med.* **28**, 490–495 (2022). 35046573 10.1038/s41591-021-01678-y PMC8767531

[113] Rogers, T. F. et al. Isolation of potent SARS-CoV-2 neutralizing antibodies and protection from disease in a small animal model. *Science* **369**, 956–963 (2020). 32540903 10.1126/science.abc7520 PMC7299280

[114] Ou, X. et al. Characterization of spike glycoprotein of SARS-CoV-2 on virus entry and its immune cross-reactivity with SARS-CoV. *Nat. Commun.* **11**, 1620 (2020). 32221306 10.1038/s41467-020-15562-9 PMC7100515

[115] Szklarczyk, D. et al. The STRING database in 2021: customizable protein–protein networks, and functional characterization of user-uploaded gene/measurement sets. *Nucleic Acids Res.* **49**, D605–D612 (2020). 10.1093/nar/gkaa1074 PMC7779004 33237311

[116] Correia, D. V. et al. Differentiation of human peripheral blood Vδ1^+^ T cells expressing the natural cytotoxicity receptor NKp30 for recognition of lymphoid leukemia cells. *Blood* **118**, 992–1001 (2011). 21633088 10.1182/blood-2011-02-339135

[117] Djaoud, Z. et al. Two alternate strategies for innate immunity to Epstein–Barr virus: one using NK cells and the other NK cells and γδ T cells. *J. Exp. Med.* **214**, 1827–1841 (2017). 28468758 10.1084/jem.20161017 PMC5460997

[118] R: A Language and Environment for Statistical Computing (R Foundation for Statistical Computing, 2021).

[119] Gu, Z. Complex heatmap visualization. *iMeta* **1**, e43 (2022). 38868715 10.1002/imt2.43 PMC10989952

[120] Gu, Z., Eils, R. & Schlesner, M. Complex heatmaps reveal patterns and correlations in multidimensional genomic data. *Bioinformatics* **32**, 2847–2849 (2016). 27207943 10.1093/bioinformatics/btw313

[121] Lex, A., Gehlenborg, N., Strobelt, H., Vuillemot, R. & Pfister, H. UpSet: visualization of intersecting sets. *IEEE Trans. Vis. Comput. Graph.* **20**, 1983–1992 (2014). 26356912 10.1109/TVCG.2014.2346248 PMC4720993

