# Supplementary material for: Interferon-mediated NK cell activation increases cytolytic activity against T follicular helper cells and limits antibody response to SARS-CoV-2
Source: Nat Immunol. 2025 Nov 21;26(12):2201–17. doi: 10.1038/s41590-025-02341-1 (PMC12643942; doi:10.1038/s41590-025-02341-1)
Supplement: Supplementary file 1 — Supplementary Figs. 1–5 and consortium members. [file 41590_2025_2341_MOESM1_ESM.pdf]

nature immunology

Supplementary information

https://doi.org/10.1038/s41590-025-02341-1

Interferon-mediated NK cell activation increases cytolytic activity against T follicular helper cells and limits antibody response to SARS-CoV-2

In the format provided by the authors and unedited

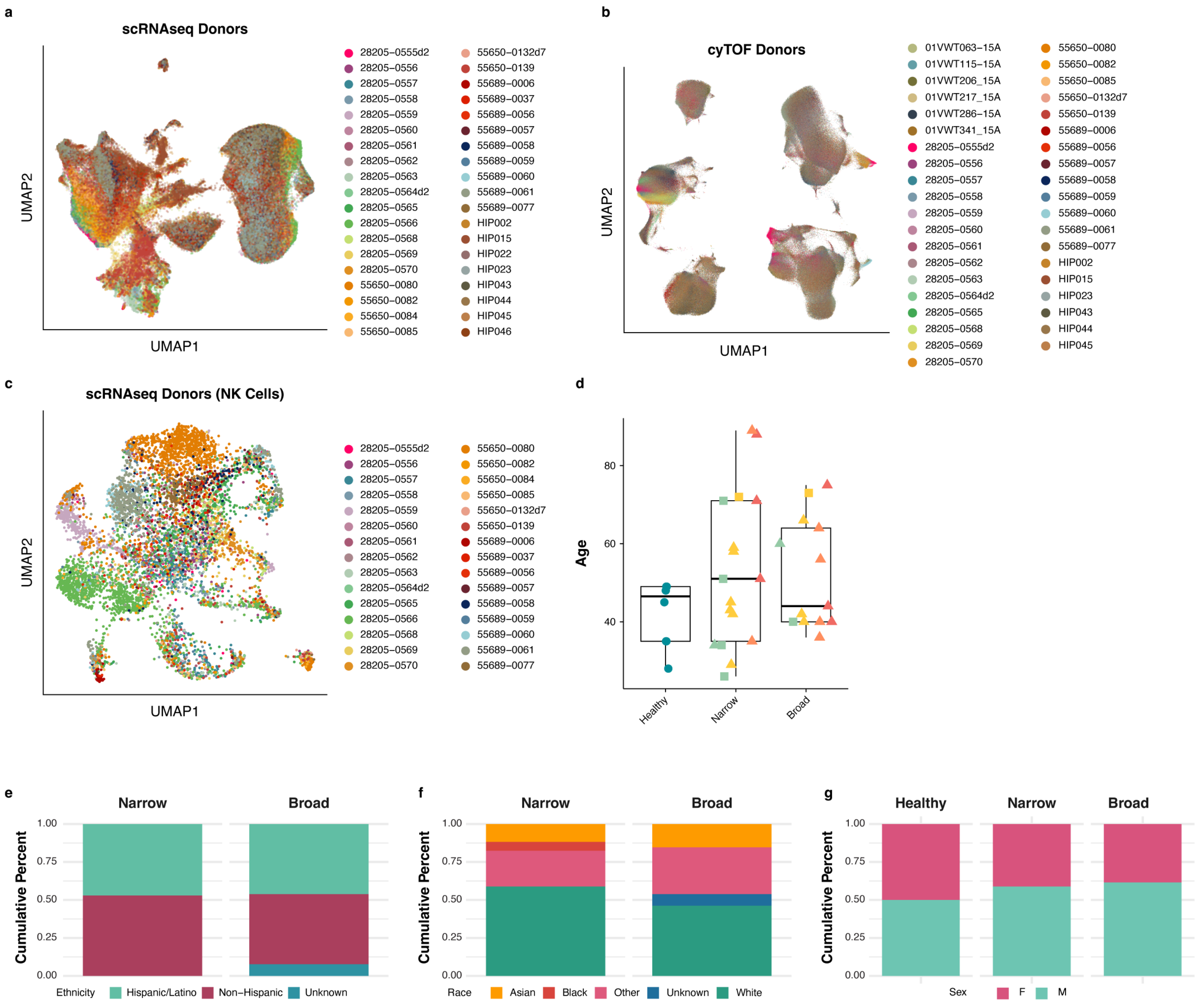

Supplemental Figure 1: Cohort Characteristics.

a. UMAP of complete scRNA-seq dataset colored by donor.

b. UMAP of complete CyTOF dataset colored by donor.

c. UMAP of NK cells from all COVID-19 subjects colored by donor

d. Box plot of age at blood draw for each donor in broad, narrow, and healthy controls colored by WHO severity score and shaped by acuity. Boxplots are drawn as median (center line), IQR (box), and 1.5x IQR (whiskers). Each point represents one donor.

e Cumulative bar plot of relative percent of reported ethnicity in broad and narrow breadth groups.

f. Cumulative bar plot of relative percent of reported race in breadth groups.

g. Cumulative bar plot of relative percent of reported sex in broad, narrow, and healthy groups. Ethnicity and race were not reported for healthy controls

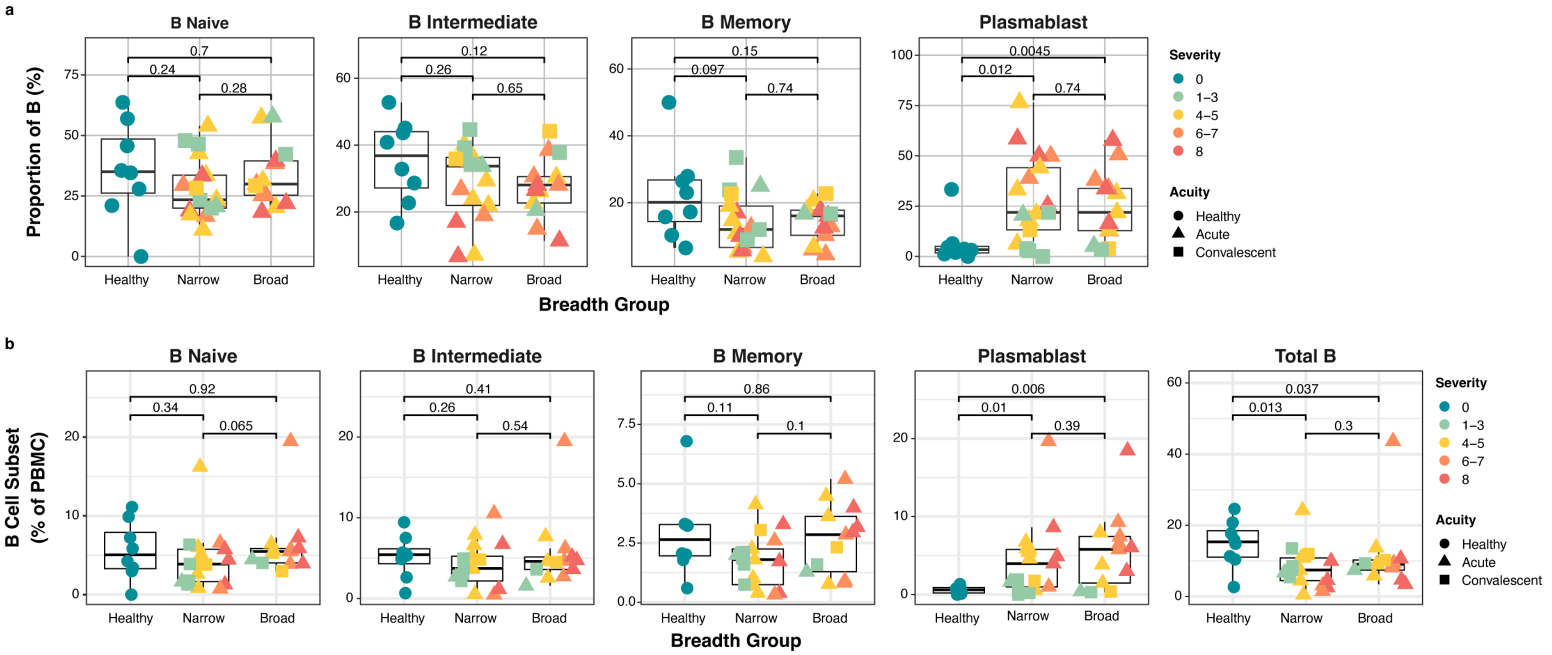

Supplemental Figure 2: B Cell Frequencies in Breadth Groups.

a. Boxplots quantifying B cell subset frequencies as a proportion of total B cells.

b. Boxplots quantifying B cell subset and total B cell frequency as a proportion of total PBMCs.

All boxplots are drawn as median (center line), IQR (box), and 1.5x IQR (whiskers), colored by peak WHO severity score, and shaped by acuity. P-values by two-sided Wilcoxon rank-sum test with Bonferroni's correction for multiple hypothesis testing. Each point represents one donor.

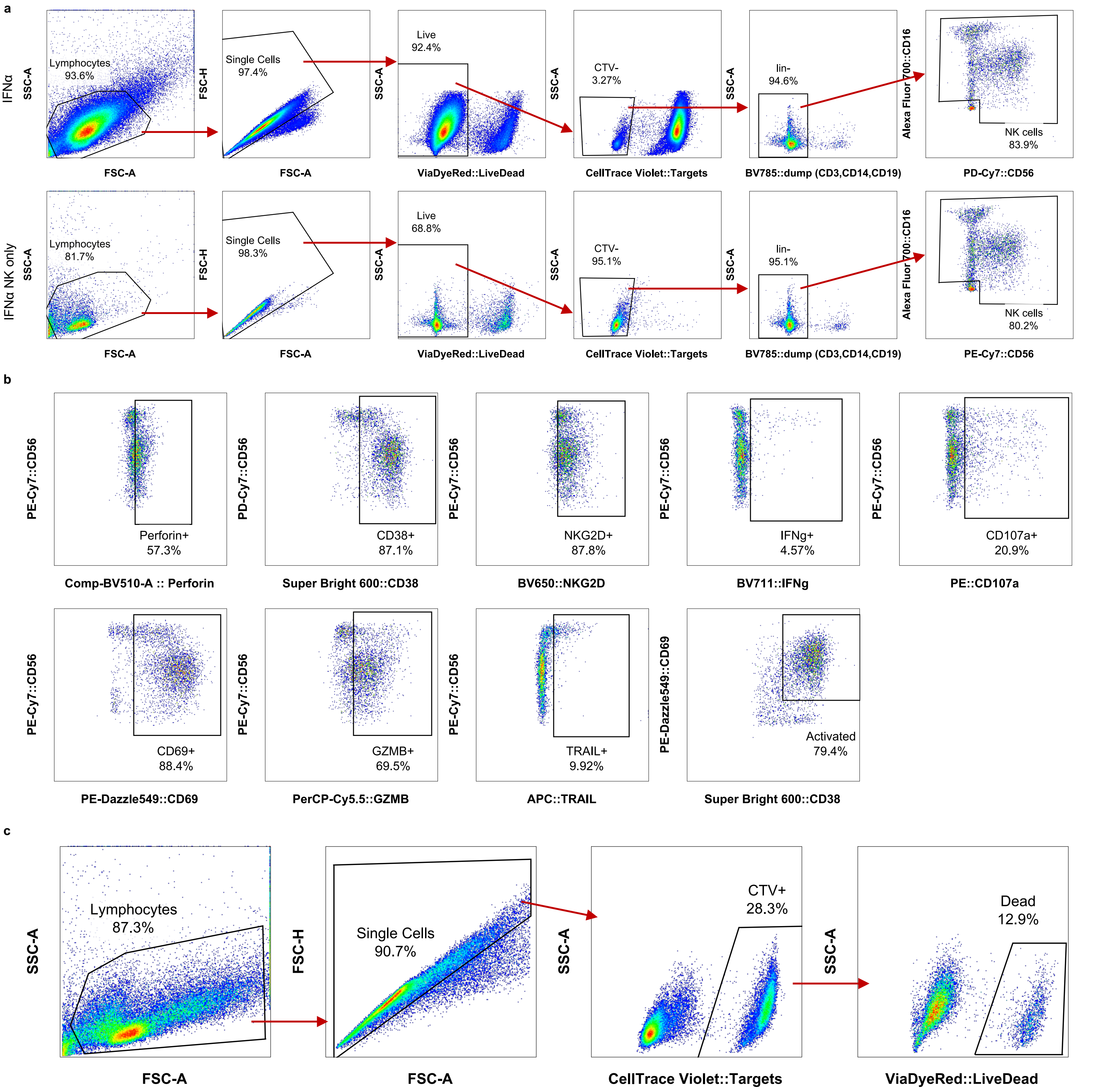

Supplementary Figure 3: Gating NK Cells and Functional Markers in co-culture with iTfh cells.

a. Representative Flow plots showing gating to identify NK cells in functional co-culture experiments for one donor in IFNα activated co-culture and mono-culture groups. lin- indicates negative gate for pooled CD3, CD19, and CD14 antibodies and target iTfh-like cells were labeled with cell trace violet (CTV).

b. Representative flow plot showing expression and percent positive gating of functional/phenotypic NK markers in co-culture experiments from one donor in IFNα group.

c. Representative flow plot showing gating to identify dead target cells in killing assay.

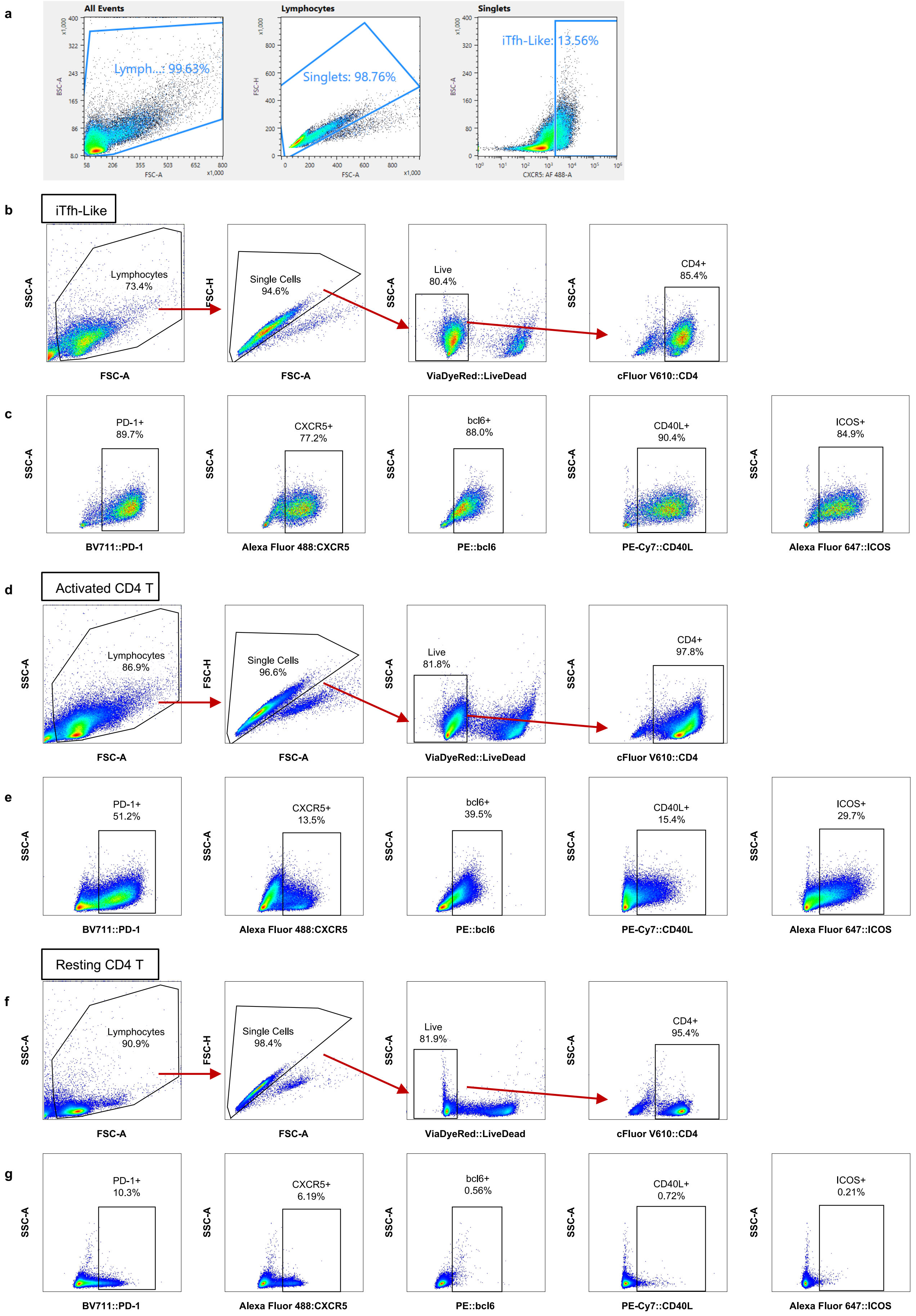

Supplementary Figure 4: Gating and Representative Flow plots of iTfh-Like and other CD4 T Cell Subsets.
a. Representative plot of sorting gates for iTfh-like cells on Sony SH800.
b. Representative gating of iTfh-like cells for analysis of phenotypic markers.
c. Representative Flow plots of Tfh phenotypic markers in Tfh like cells. Each plot from CD4+ in b.
d. Representative gating activated CD4 T cells for analysis of phenotypic markers.
e. Representative Flow plots of Tfh phenotypic markers in activated CD4 T cells. Each plot from CD4+ in d.
f. Representative gating of resting CD4 T cells for analysis of phenotypic markers.
g. Representative Flow plots of Tfh phenotypic markers in resting CD4 T cells. Each plot from CD4+ in f.

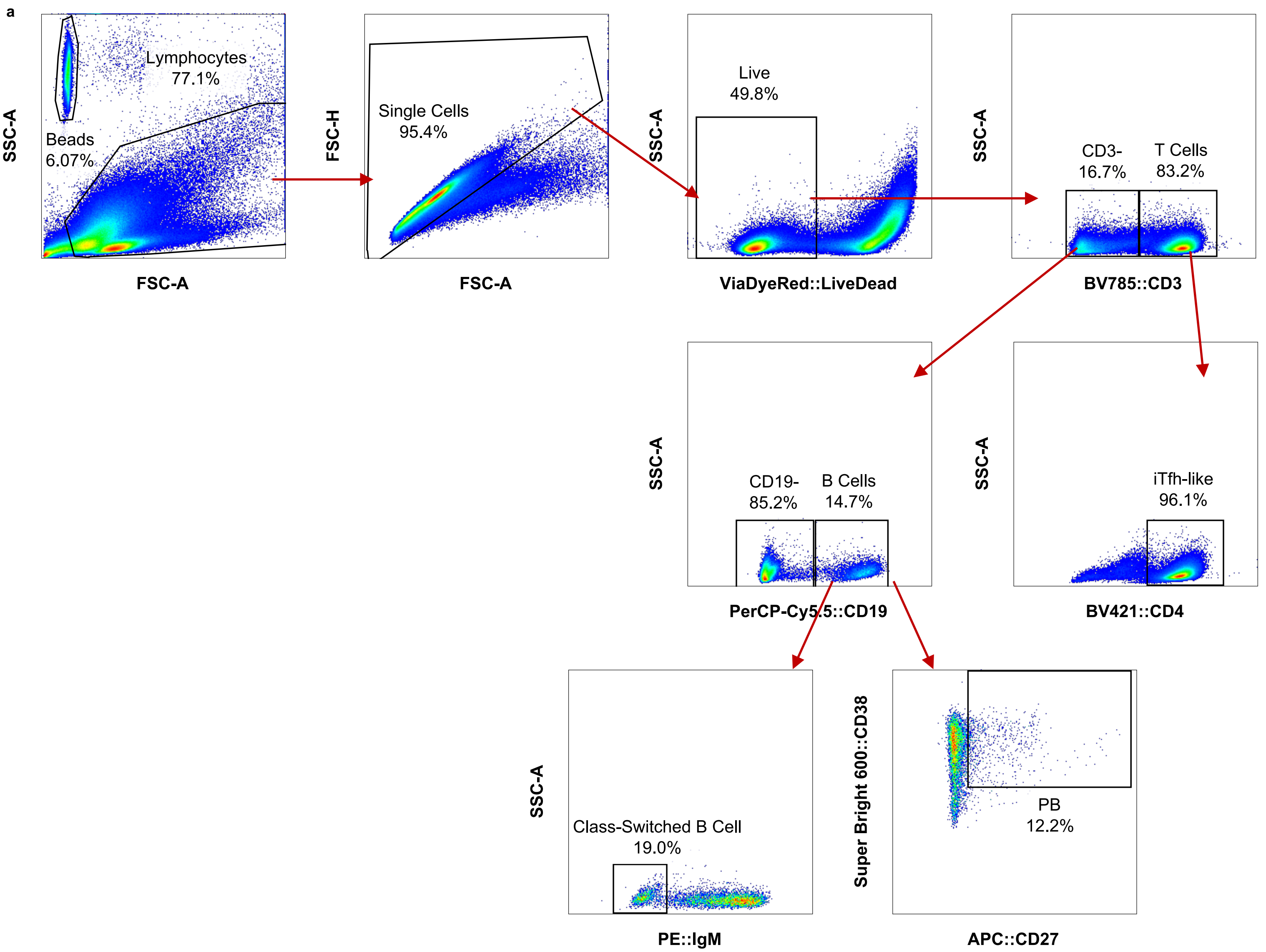

Supplementary Figure 5: Gating and Representative Flow plots of iTfh-like, B, NK Co-Culture.
a. Representative plot of gating to evaluate beads, iTfh-like, B, Class-switched B, and PB cell numbers after co-culture for 6 days.

Stanford COVID-19 Biobank members:

Thanmayi Ranganath, Nancy Q. Zhao, Aaron J. Wilk, Rosemary Vergara, Julia L. McKechnie, Lauren de la Parte, Kathleen Whittle Dantzler, Maureen Ty, Nimish Kathale, Giovanny J. Martínez-Colón, Arjun Rustagi, Geoff Ivison, Ruoxi Pi, Madeline J. Lee, Rachel Brewer,Taylor Hollis, Andrea Baird, Michele Ugur, Michal Tal, Drina Bogusch, Georgie Nahass, Kazim Haider, Kim Quyen Thi Tran, Laura Simpson, Hena Din, Jonasel Roque, Rosen Mann, Iris Chang, Evan Do, Andrea Fernandes, Shu-Chen Lyu, Wenming Zhang, Monali Manohar, James Krempski, Anita Visweswaran, Elizabeth J. Zudock, Kathryn Jee, Komal Kumar, Jennifer A. Newberry, James V. Quinn, Donald Schreiber, Euan A. Ashley, Catherine A. Blish, Andra L. Blomkalns, Kari C. Nadeau, Ruth O'Hara, Angela J. Rogers, Samuel Yang.
