# Supplementary material for: Interferon-mediated NK cell activation increases cytolytic activity against T follicular helper cells and limits antibody response to SARS-CoV-2
Source: Nat Immunol. 2025 Nov 21;26(12):2201–17. doi: 10.1038/s41590-025-02341-1 (PMC12643942; doi:10.1038/s41590-025-02341-1)
Supplement: Supplementary file 2 — Reporting Summary [file 41590_2025_2341_MOESM2_ESM.pdf]

nature portfolio

Corresponding author(s): Catherine A. Blish

Last updated by author(s): Oct 3, 2025

Reporting Summary

Nature Portfolio wishes to improve the reproducibility of the work that we publish. This form provides structure for consistency and transparency in reporting. For further information on Nature Portfolio policies, see our Editorial Policies and the Editorial Policy Checklist.

Statistics

For all statistical analyses, confirm that the following items are present in the figure legend, table legend, main text, or Methods section.

n/a	Confirmed	
☐	☒	The exact sample size (*n*) for each experimental group/condition, given as a discrete number and unit of measurement
☐	☒	A statement on whether measurements were taken from distinct samples or whether the same sample was measured repeatedly
☐	☒	The statistical test(s) used AND whether they are one- or two-sided<br>*Only common tests should be described solely by name; describe more complex techniques in the Methods section.*
☐	☒	A description of all covariates tested
☐	☒	A description of any assumptions or corrections, such as tests of normality and adjustment for multiple comparisons
☐	☒	A full description of the statistical parameters including central tendency (e.g. means) or other basic estimates (e.g. regression coefficient) AND variation (e.g. standard deviation) or associated estimates of uncertainty (e.g. confidence intervals)
☐	☒	For null hypothesis testing, the test statistic (e.g. *F*, *t*, *r*) with confidence intervals, effect sizes, degrees of freedom and *P* value noted<br>*Give P values as exact values whenever suitable.*
☒	☐	For Bayesian analysis, information on the choice of priors and Markov chain Monte Carlo settings
☒	☐	For hierarchical and complex designs, identification of the appropriate level for tests and full reporting of outcomes
☐	☒	Estimates of effect sizes (e.g. Cohen's *d*, Pearson's *r*), indicating how they were calculated

Our web collection on statistics for biologists contains articles on many of the points above.

Software and code

Policy information about availability of computer code

Data collection	Neutralization assays and ELISA read using Promega Glomax plate reader (Promega; GM3000).<br>Spectral unmixing was performed in Cytek Spectroflo Software using UltraComp eBeads Plus Compensation Beads (Thermo Scientific; 01-3333-42) as single color controls. Unmixed FCS files were imported to FlowJo (V10.10.0).<br>QuantStudio 3 Real-Time PCR System was used to quantify transcript levels (Thermo Fisher; A28567)
Data analysis	Statistics and visualizations were performed in the open source software R (V4.2.2) and ggolot2. Seurat (V4.4.0) was used to normalize and scale scRNA-seq data as well as differential expression and gene module scoring. DESeq2 (V1.38.3) was used for pseudobulk correlation analysis. Bionet(V1.58.0) was used for network analysis. MulitnichenetR (V1.0.1) was used for cell-cell communication. CytoTRACE2 (V1.0.0) was used for cell potency analysis. FlowJo V10.10.0 was used to analyze flow cytometry data. Code used to generate figures will be available at https://github.com/BlishLab/SARSCoV2_Antibody_Breadth upon publication.

For manuscripts utilizing custom algorithms or software that are central to the research but not yet described in published literature, software must be made available to editors and reviewers. We strongly encourage code deposition in a community repository (e.g. GitHub). See the Nature Portfolio guidelines for submitting code & software for further information.

Data

Policy information about availability of data

All manuscripts must include a data availability statement. This statement should provide the following information, where applicable:

- Accession codes, unique identifiers, or web links for publicly available datasets
- A description of any restrictions on data availability
- For clinical datasets or third party data, please ensure that the statement adheres to our policy

scRNAseq and CyTOF data was originally published in Wilk et al. JExMed. 2021. FCS files (CyTOF) with de-identified metadata supporting this publication are available at ImmPort (https://www. immport.org) under study accession no. SDY1708. Data from scRNA-seq have been deposited with the Gene Expression Omnibus under accession no. GSE174072. FCS flow cytometry files from in vitro assays are deposited at CytoBank Community Experiment IDs: 123058.

Research involving human participants, their data, or biological material

Policy information about studies with human participants or human data. See also policy information about sex, gender (identity/presentation), and sexual orientation and race, ethnicity and racism.

Reporting on sex and gender	Biological sex was self reported
Reporting on race, ethnicity, or other socially relevant groupings	Race and ethnicity was self reported
Population characteristics	NA
Recruitment	Samples were obtained from the Stanford COVID-19 Biobank with informed consent under protocols approved by the Stanford University Institutional Review Board.
Ethics oversight	NA

Note that full information on the approval of the study protocol must also be provided in the manuscript.

Field-specific reporting

Please select the one below that is the best fit for your research. If you are not sure, read the appropriate sections before making your selection.

☒ Life sciences ☐ Behavioural & social sciences ☐ Ecological, evolutionary & environmental sciences

For a reference copy of the document with all sections, see nature.com/documents/nr-reporting-summary-flat.pdf

Life sciences study design

All studies must disclose on these points even when the disclosure is negative.

Sample size	Sample size for in-vitro experiments was chosen to achieve at least 80% power and a two-sided type I error of 5%.
Data exclusions	scRNA-seq was qc-ed as described in Wilk et al. 2021. Patients from Wilk et al. 2021. without matched serum tested for breadth were excluded
Replication	All in-vitro experiments were replicated successfully
Randomization	NA
Blinding	NA

Reporting for specific materials, systems and methods

We require information from authors about some types of materials, experimental systems and methods used in many studies. Here, indicate whether each material, system or method listed is relevant to your study. If you are not sure if a list item applies to your research, read the appropriate section before selecting a response.

Materials & experimental systems

n/a	Involved in the study
☐	☒ Antibodies
☐	☒ Eukaryotic cell lines
☒	☐ Palaeontology and archaeology
☒	☐ Animals and other organisms
☒	☐ Clinical data
☒	☐ Dual use research of concern
☒	☐ Plants

Methods

n/a	Involved in the study
☒	☐ ChIP-seq
☐	☒ Flow cytometry
☒	☐ MRI-based neuroimaging

Antibodies

Antibodies used

See Supplementary Material for cyTOF panel from Wilk et al. 2021
Perforin Brilliant Violet 510 dG9 BioLegend 308120
NKG2D Brilliant Violet 650 1D11 BD 563408
CD38 Superbright 600 HB7 Thermo Scientific 63-0388-42
IFNg Brilliant Violet 711 4S.B3 BioLegend 502540
CD3 Brilliant Violet 785 OKT3 BioLegend 317330
CD14 Brilliant Violet 785 M5E2 BioLegend 301840
CD19 Brilliant Violet 785 HIB19 BioLegend 302240
CD107a PE H4A3 BioLegend 328608
CD69 Dazzle FN50 BioLegend 310942
Granzyme B PerCP-Cy5.5 QA16A02 BioLegend 372212
CD56 PE-Cy7 HCD56 BioLegend 318318
TRAIL/CD253 APC RIK-2 BioLegend 308210
CD16 Alexa Fluor 700 2G8 BioLegend 302026
CD4 cFluor V610 SK3 Cytek Biosciences R7-20073
PD1 Brilliant Violet 711 EH12.2H7 BioLegend 329928
CXCR5/CD185 Alexa Fluor 488 J252D4 BioLegend 356912
ICOS Alexa Fluor 647 DX29 BD 562834
Viability ViaDye Red NA Cytek Biosciences R7-60008
CD40L/CD154 PE-Cy7 24-31 Biolegend 310832
BCL6 PE K112-91 BD 561522
B7-H6 APC 875001 R&D System FAB7144A
ULBP1 APC 170818 R&D System FAB1380A
ULBP2-5-6 APC 165903 R&D System FAB1298A
ULBP3 APC 166510 R&D System FAB1517A
MICA APC 159227 R&D System FAB1300A-100
MICB APC 236511 R&D System FAB1599A-100
CD48 APC BJ40 Biolegend 336714
IgG1 APC 11711 R&D System IC002A
IgG2a APC 20102 R&D System IC003A
IgG2b APC 133303 R&D System IC0041A
IgG1ϰ APC MOPC-21 Biolegend 400121
CX3CR1 BV421 2A9-1 Biolegend 341620
CD4 BV421 RPA-T4 Biolegend 300532
IgM PE MHM-88 Biolegend 314508
CD19 PerCP-cy5.5 HIB19 Biolegend 302230
CD27 APC O323 Cytek Biosciences 20-0279-T100
NKG2D NA 1D11 Biolegend 320802
NKp30 NA 210845 R&D System MAB1849-SP

Validation

All antibodies were commercially available and validated by vendors. We also tested antibodies on antigen negative and positive cells and titered antibodies stain antigen positive cells without background signal in antigen-negative cells and validated fluorescence minus one samples for all fluors in all panels. Gates were set using unstained samples.

Eukaryotic cell lines

Policy information about cell lines and Sex and Gender in Research

Cell line source(s)

HeLa Cell line stably overexpressing ACE2 and TMPRSS2 produced as described in Rogers et al. 2020. 293T cells (ATCC).

Authentication

ACE2 epression was validated on HeLa-ACE2TMPRSS2 by our collaborators. The original cell lines were not authenticated beyond purchasing them from vendors or acquiring them through collaborators.

Mycoplasma contamination

Cell lines were negative for mycoplasma

Commonly misidentified lines
(See ICLAC register)

NA

Plants

Seed stocks: NA

Novel plant genotypes: NA

Authentication: NA

Flow Cytometry

Plots

Confirm that:

☒ The axis labels state the marker and fluorochrome used (e.g. CD4-FITC).

☒ The axis scales are clearly visible. Include numbers along axes only for bottom left plot of group (a 'group' is an analysis of identical markers).

☒ All plots are contour plots with outliers or pseudocolor plots.

☒ A numerical value for number of cells or percentage (with statistics) is provided.

Methodology

Sample preparation: Samples were isolated from primary healthy human PBMCs as described in methods. If used, 4 hrs before the end of co-culture Brefeldin A (eBioscience; 00-4506-51), Monensin (eBioscience;00-4501-51), and anti-CD107a PE was added to the culture, followed by centrifugation at 1000 RPM for 1 min. When co-cultures or other samples were ready, cells were washed in FACS twice and resuspended in ViaDye Red at 1:6000 dilution in PBS. After 30 min at room temperature, cells were washed twice with FACS and resuspended in a cocktail of surface antibodies in 1X Brilliant Stain Buffer (BD; 563794) (if needed) diluted with FACS for another 30 min at room temperature. After surface stain, cells were washed twice in FACS and fixed in 2% paraformaldehyde for 10 minutes at RT. If needed, cells were then washed twice in 1X permeabilization buffer (eBioscience; 00-8333-56) and stained with intracellular antibodies in 1X Brilliant Stain Buffer and 1X permeabilization buffer diluted in water for 30 min at RT. Cells were then washed twice with permeabilization buffer, resuspended in FACS and analyzed on Cytek Aurora flow cytometer.

Instrument: 3 laser Cytek Aurora

Software: Spectral unmixing was performed in Cytek Spectroflo Software using UltraComp eBeads Plus Compensation Beads (Thermo Scientific; 01-3333-42) as single color controls. FlowJo V10.10.0 was used to analyze flow cytometry data

Cell population abundance: Purity was analyzed by flow cytometry after sorting

Gating strategy: SSC/FSC was used to identify lymphocytes. FSC was used to identify singlets. ViaDye Red negative cells were considered live. NK cells were identified by lack of expression of CD3/CD14/CD19 and CD56/CD16 expression. iTfh-like cells were considered CD3+CD4+CXCR5+. Target cells were identified by celltrace violet labeling. All marker gating is found in Supplementary Information

☒ Tick this box to confirm that a figure exemplifying the gating strategy is provided in the Supplementary Information.
